# Supplementary material for: Mutational constraint analysis workflow for overlapping short open reading frames and genomic neighbors
Source: BMC Genomics. 2025 Mar 14;26:254. doi: 10.1186/s12864-025-11444-w (PMC11909976; doi:10.1186/s12864-025-11444-w)
Supplement: Supplementary file 1 — Supplementary Material 1 [file 12864_2025_11444_MOESM1_ESM.docx]

**Mutational Constraint Analysis Workflow for Overlapping Short Open Reading Frames and Genomic Neighbours (Appendix)**

Martin Danner^1,2^, Matthias Begemann^1^, Florian Kraft^1^, Miriam Elbracht^1^, Ingo Kurth^1^, Jeremias Krause^1*^

^1^Institute for human genetics and genomic medicine, Medical Faculty, RWTH Aachen University Hospital, Pauwelsstrasse 30, Aachen, 52074, North-Rhine-Westphalia, Germany.

^2^scieneers GmbH, Kantstraße 1a, Karlsruhe, 76137, Baden-Wuerttemberg, Germany.

**Unravelling the constraint of the individual sORF classes**

Additionally, to visualising the combined constraint values across all sORF classes, we visualised and analysed the individual sORF classes on their own and provide the results of this analysis in this supplementary material.

**SNVOEUF distributions for the individual sORF classes**

We began this Analysis by listing the individual SNVOEUF distributions of the sORFs, as calculated using the gnomAD genome data.


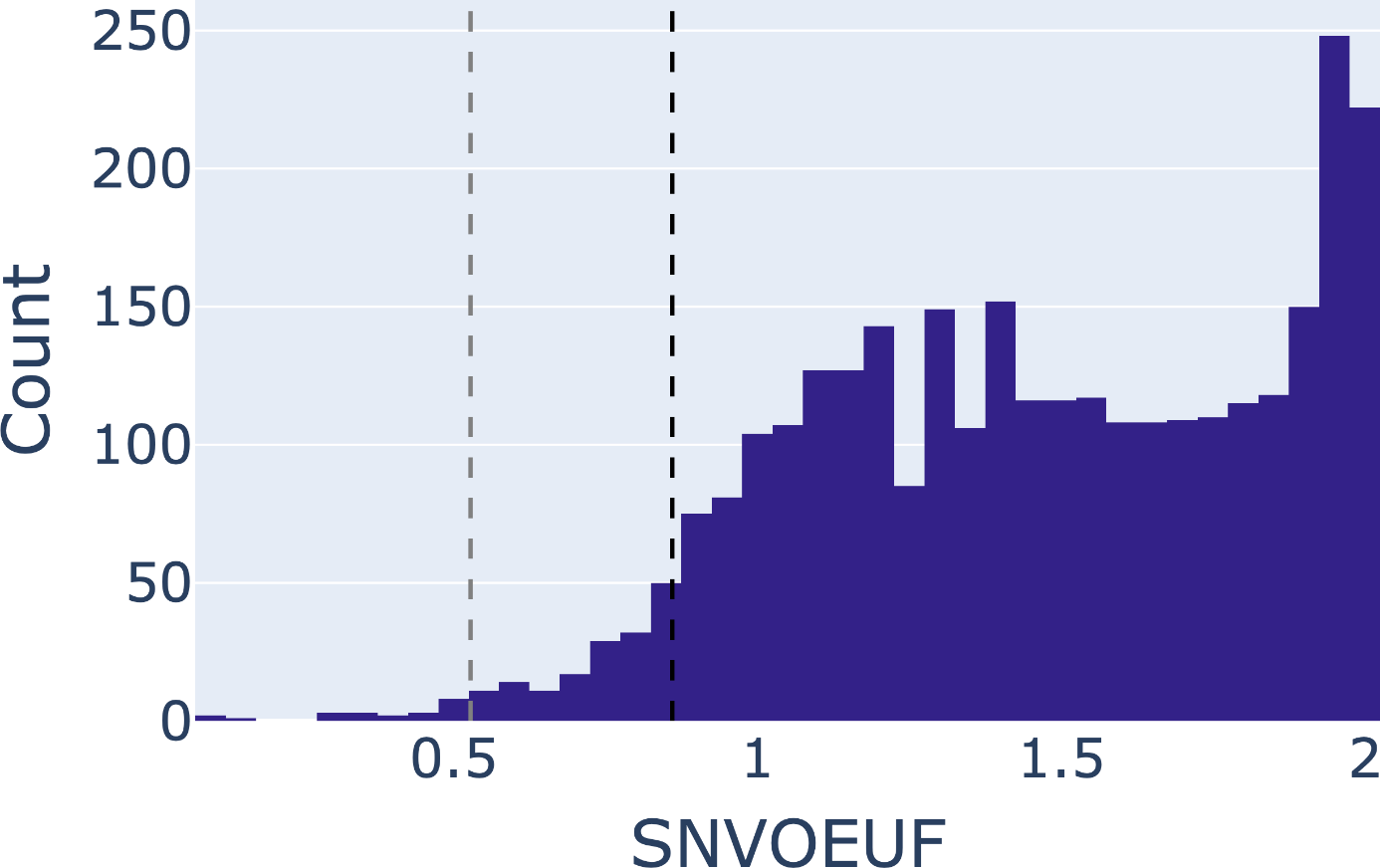


**Supplementary Figure 1**: SNVOEUF distribution for the uORF sORF class, calculated using the gnomAD genome data. Decile comparison was performed as introduced previously. 0.86 (black vertical line) is the SNVOEUF value of the mane select transcripts of canonical genes. 0.53 (grey vertical line) is the SNVOEUF value of UTRs from mane select transcripts.


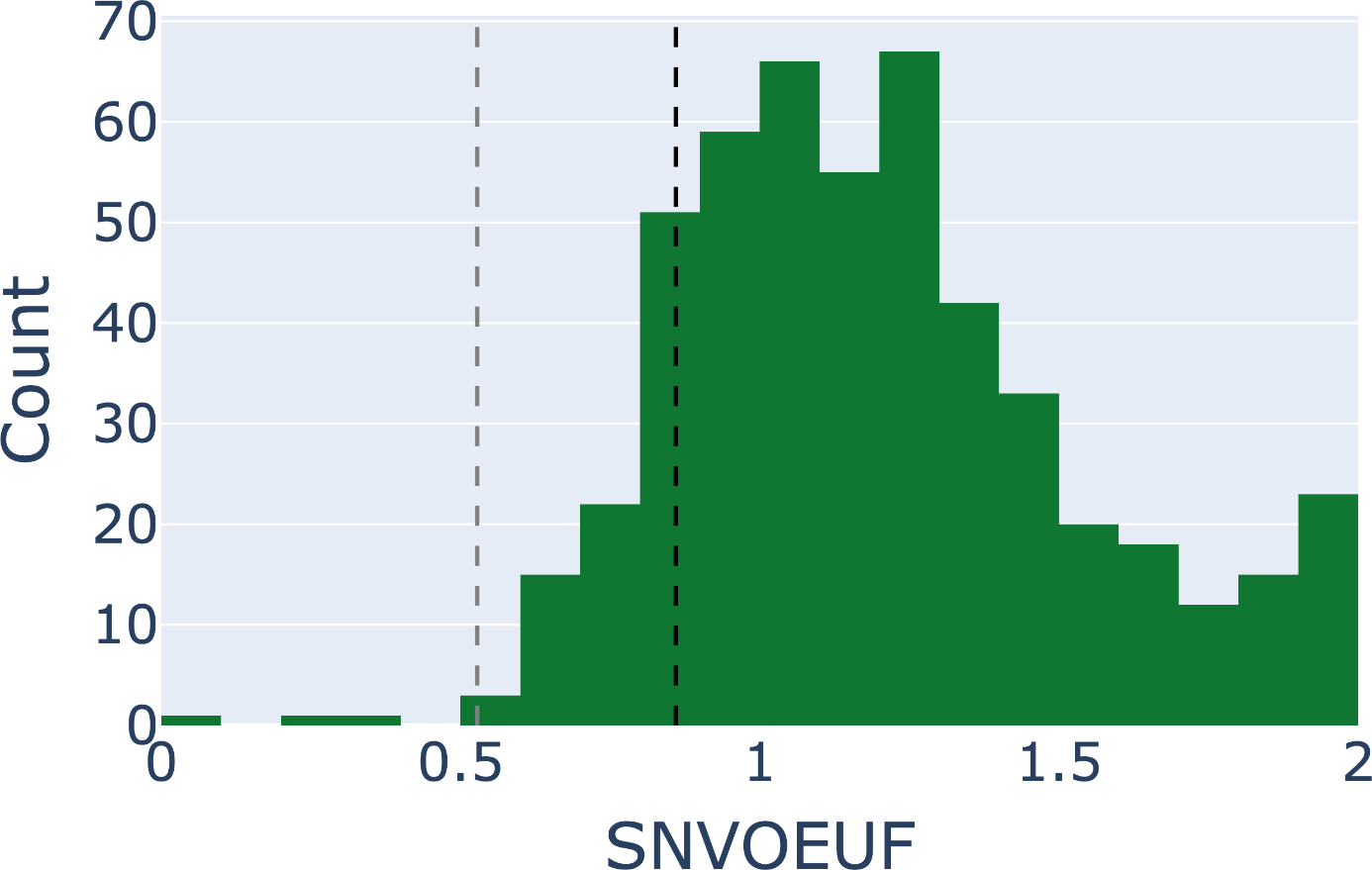


**Supplementary Figure 2:** SNVOEUF distribution for the dORF sORF class, calculated using the gnomAD genome data. Decile comparison was performed as introduced previously. 0.86 (black vertical line) is the SNVOEUF value of the mane select transcripts of canonical genes. 0.53 (grey vertical line) is the SNVOEUF value of UTRs from mane select transcripts.


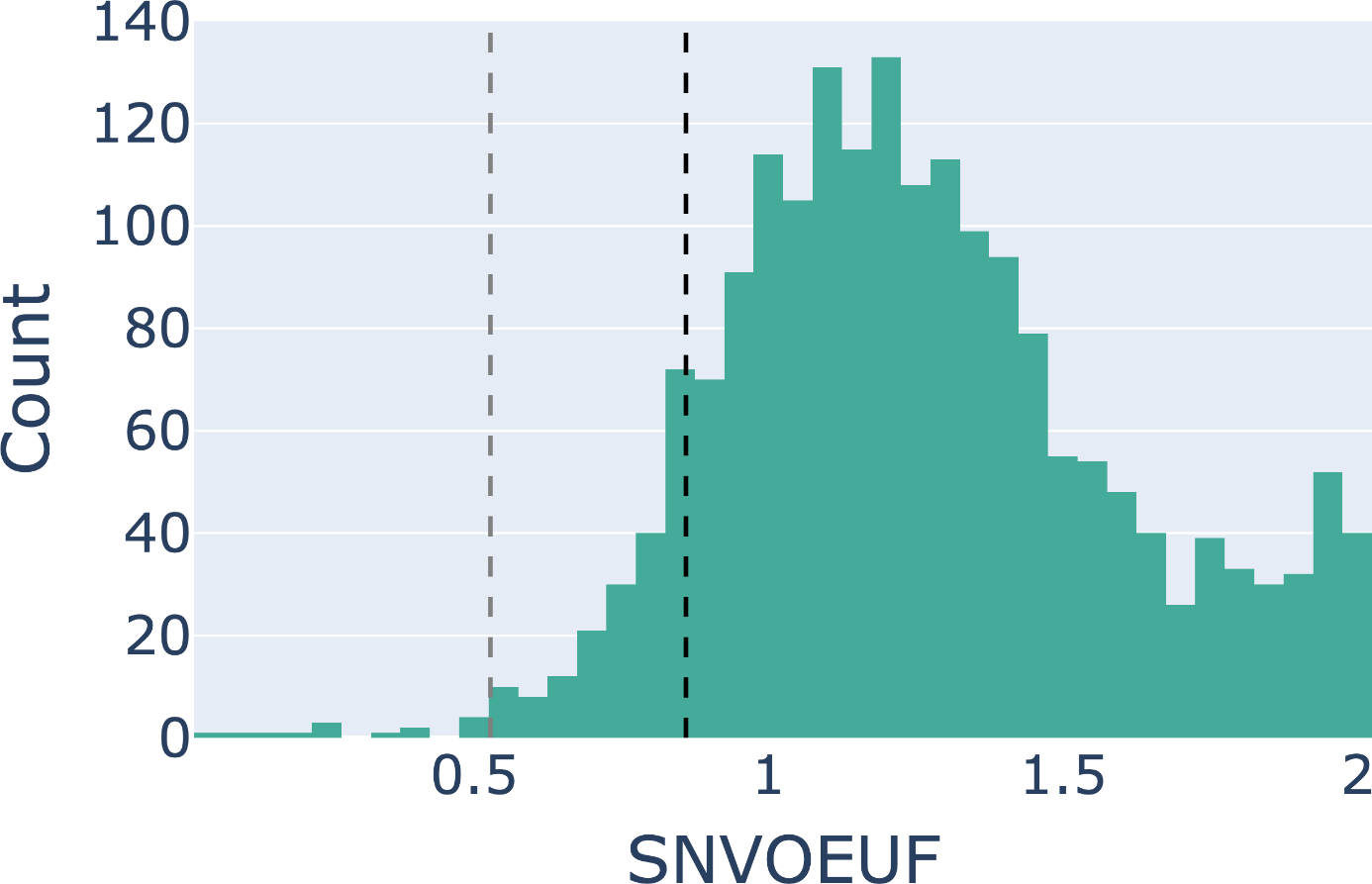


**Supplementary Figure 3:** SNVOEUF distribution for the sORF class located on lncRNAs, calculated using the gnomAD genome data. Decile comparison was performed as introduced previously. 0.86 (black vertical line) is the SNVOEUF value of the mane select transcripts of canonical genes. 0.53 (grey vertical line) is the SNVOEUF value of UTRs from mane select transcripts.


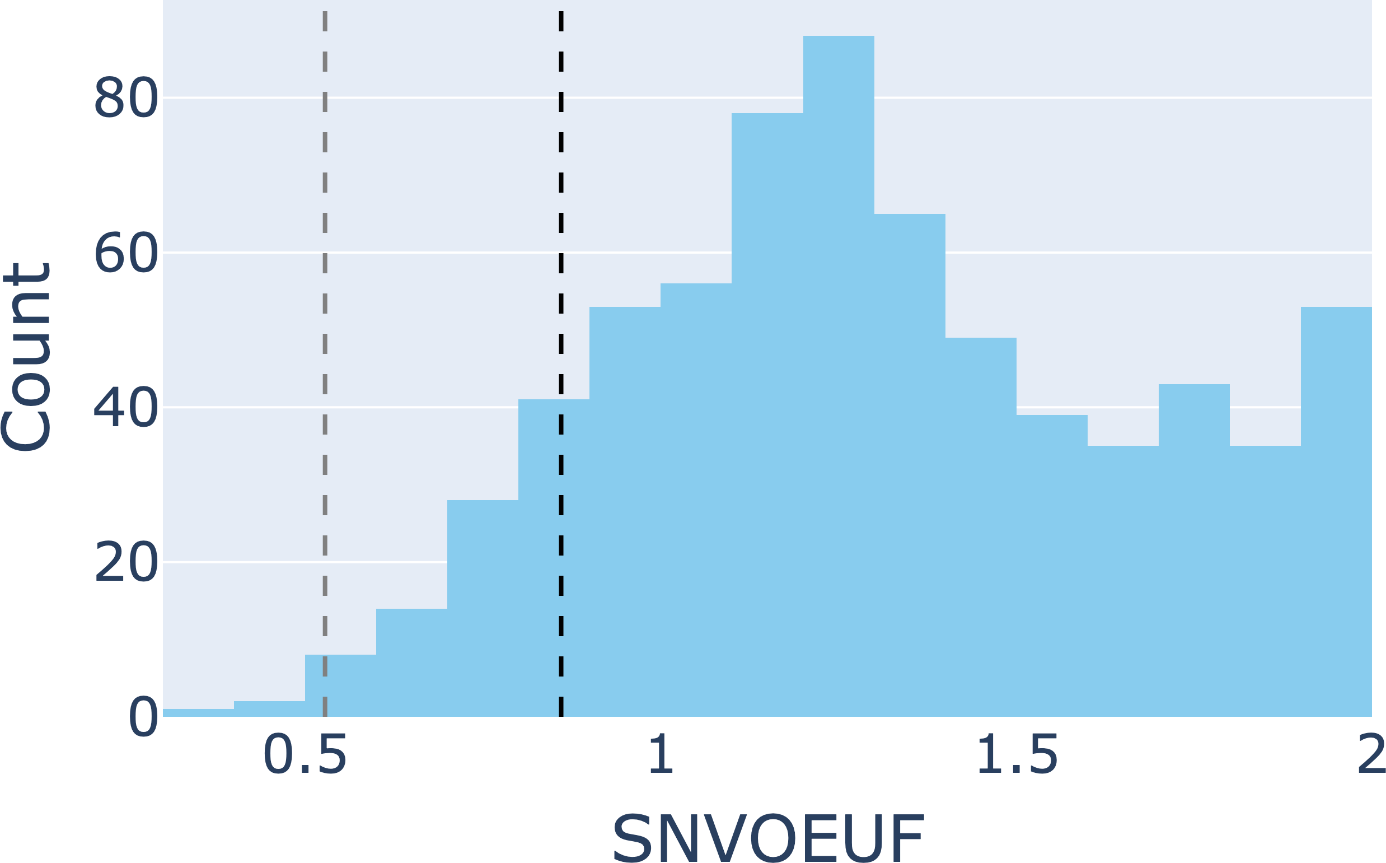


**Supplementary Figure 4**: SNVOEUF distribution for the uoORF sORF class, calculated using the gnomAD genome data. Decile comparison was performed as introduced previously. 0.86 (black vertical line) is the SNVOEUF value of the mane select transcripts of canonical genes. 0.53 (grey vertical line) is the SNVOEUF value of UTRs from mane select transcripts.


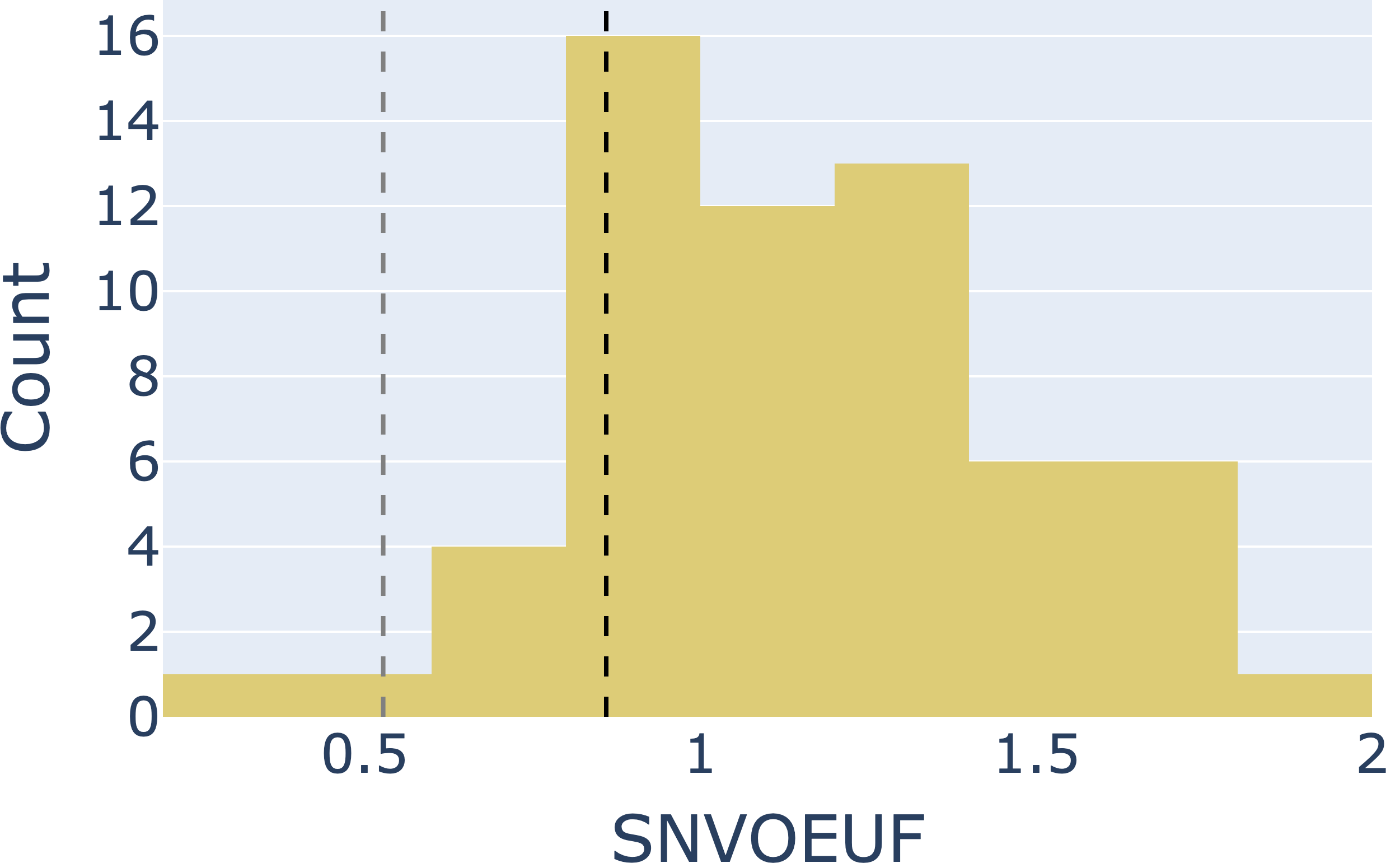


**Supplementary Figure 5:** SNVOEUF distribution for the doORF sORF class, calculated using the gnomAD genome data. Decile comparison was performed as introduced previously. 0.86 (black vertical line) is the SNVOEUF value of the mane select transcripts of canonical genes. 0.53 (grey vertical line) is the SNVOEUF value of UTRs from mane select transcripts.


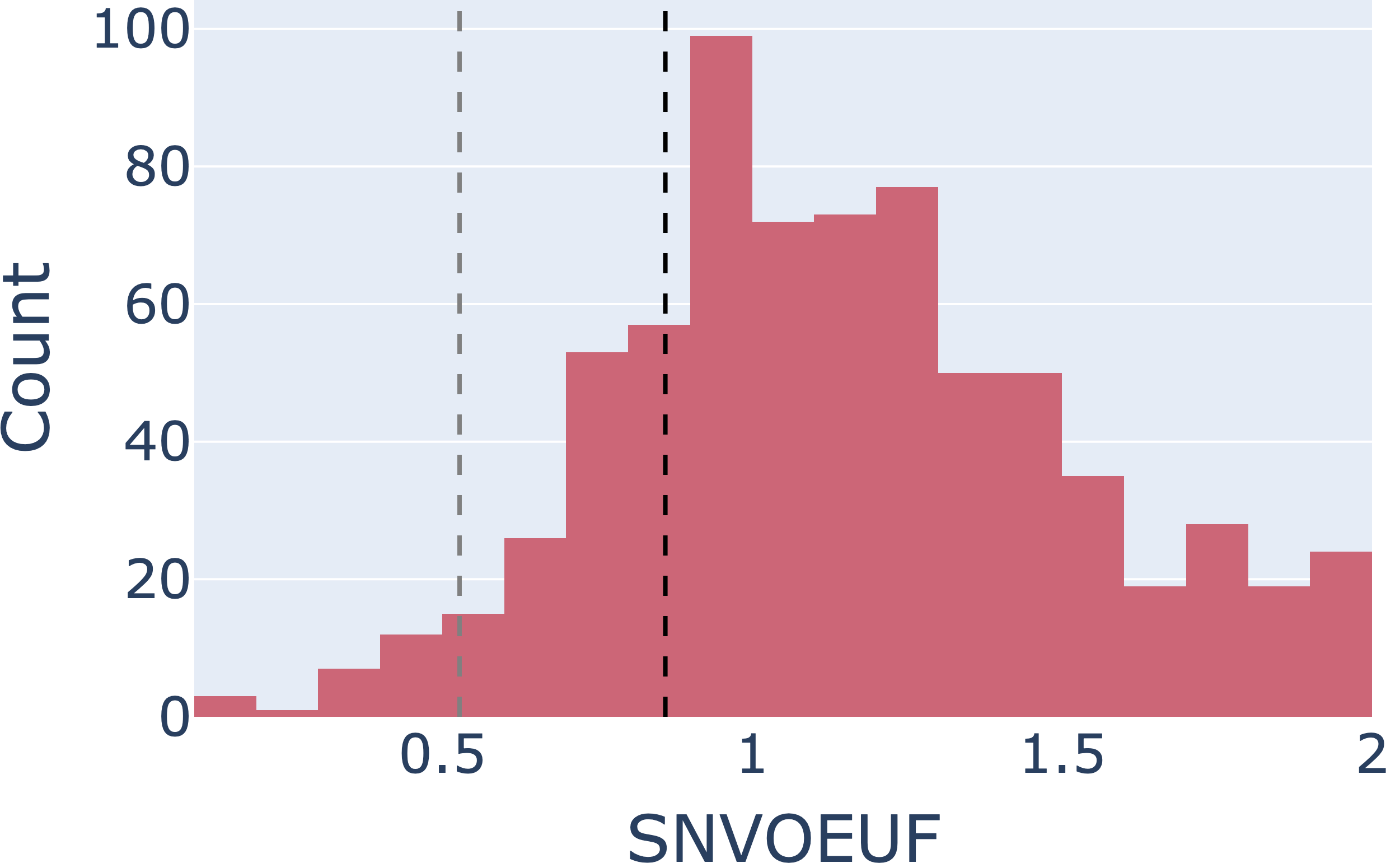


**Supplementary Figure 6:** SNVOEUF distribution for the intORF sORF class, calculated using the gnomAD genome data. Decile comparison was performed as introduced previously. 0.86 (black vertical line) is the SNVOEUF value of the mane select transcripts of canonical genes. 0.53 (grey vertical line) is the SNVOEUF value of UTRs from mane select transcripts.

**MOEUF distributions for the individual sORF classes (genome data)**

Subsequently we listed the MOEUF distributions for the individual sORF classes using the gnomAD genome data.


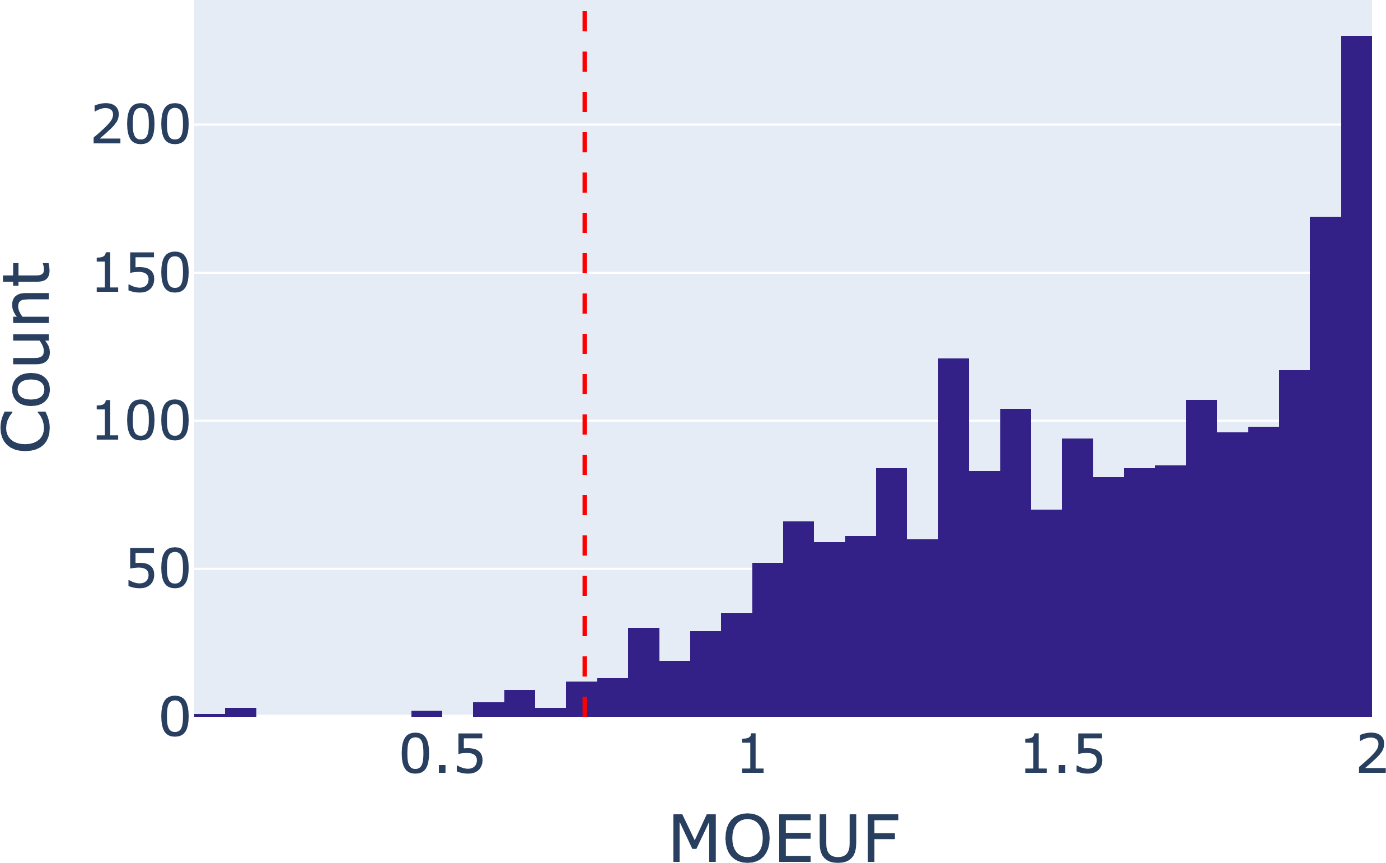


**Supplementary Figure 7:** MOEUF distribution for the uORF sORF class, calculated using the gnomAD genome data. Decile comparison was performed as introduced previously. The red vertical line (0.73) marks the most constrained decile of the MOEUF value of OEUF scores calculated for the MANE select transcripts of the canonical genes using the gnomAD genome data.


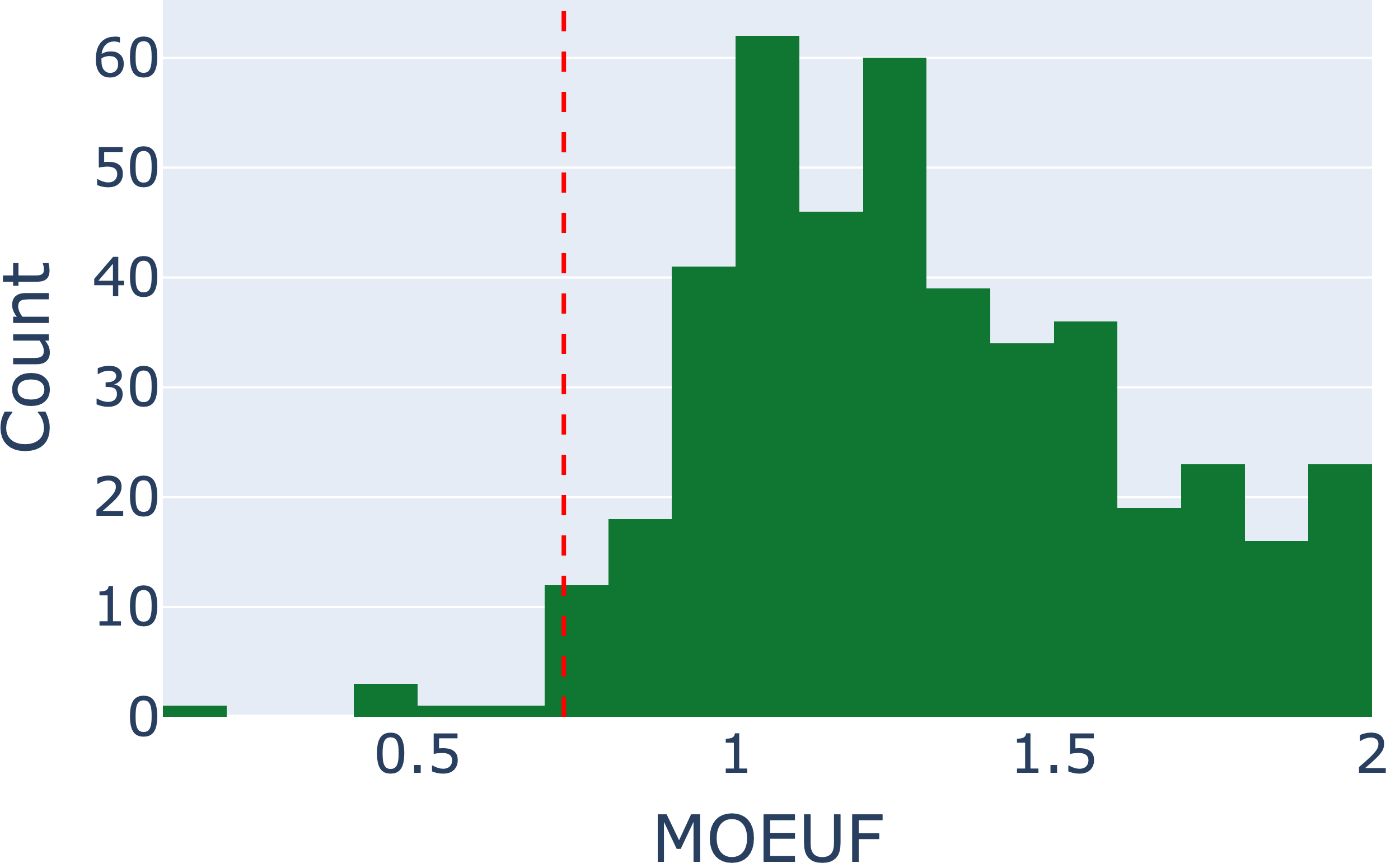


**Supplementary Figure 8:** MOEUF distribution for the dORF sORF class, calculated using the gnomAD genome data. Decile comparison was performed as introduced previously. The red vertical line (0.73) marks the most constrained decile of the MOEUF value of OEUF scores calculated for the MANE select transcripts of the canonical genes using the gnomAD genome data.


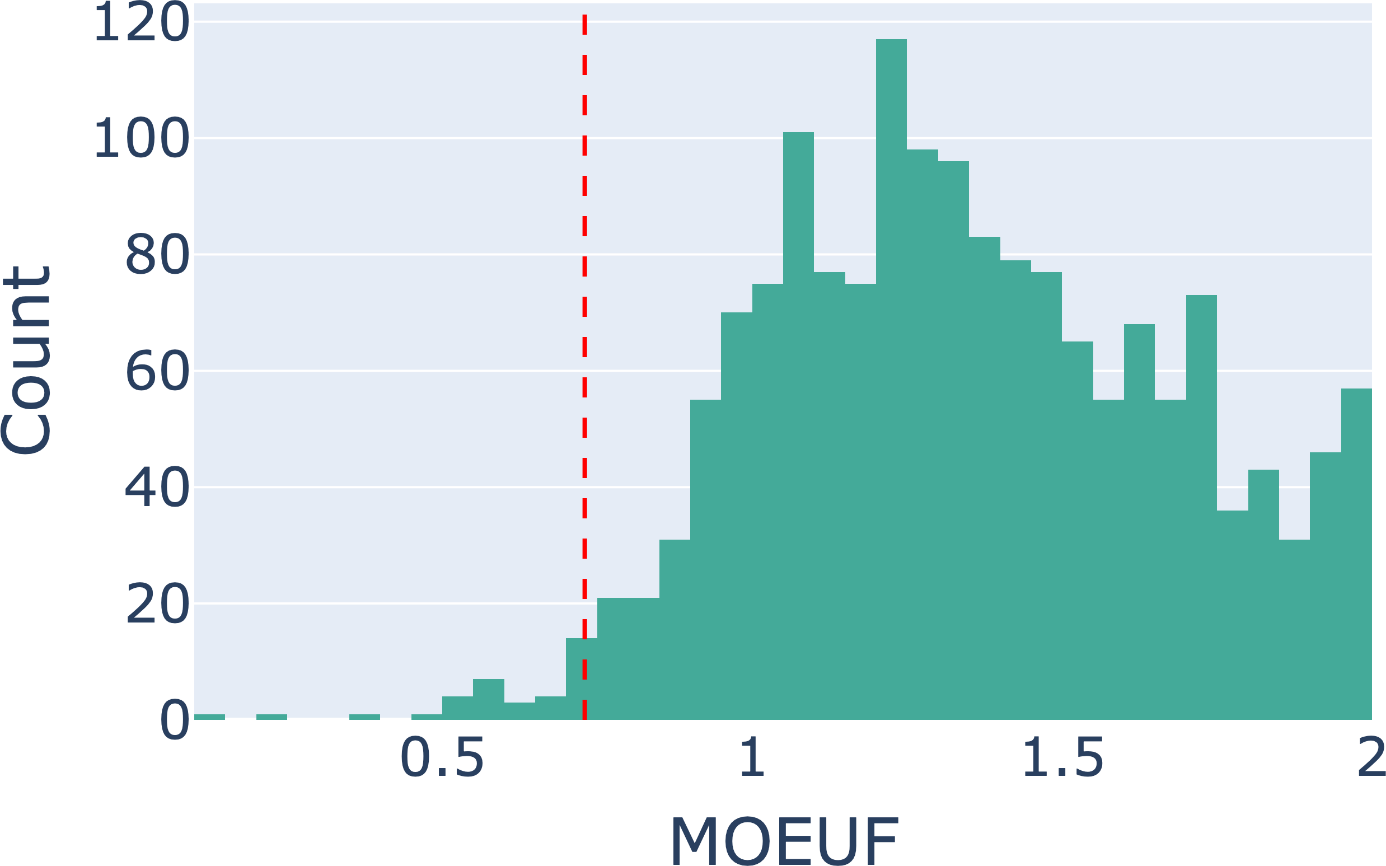


**Supplementary Figure 9:** MOEUF distribution for the sORFs located on lncRNAs, calculated using the gnomAD genome data. Decile comparison was performed as introduced previously. The red vertical line (0.73) marks the most constrained decile of the MOEUF value of OEUF scores calculated for the MANE select transcripts of the canonical genes using the gnomAD genome data.


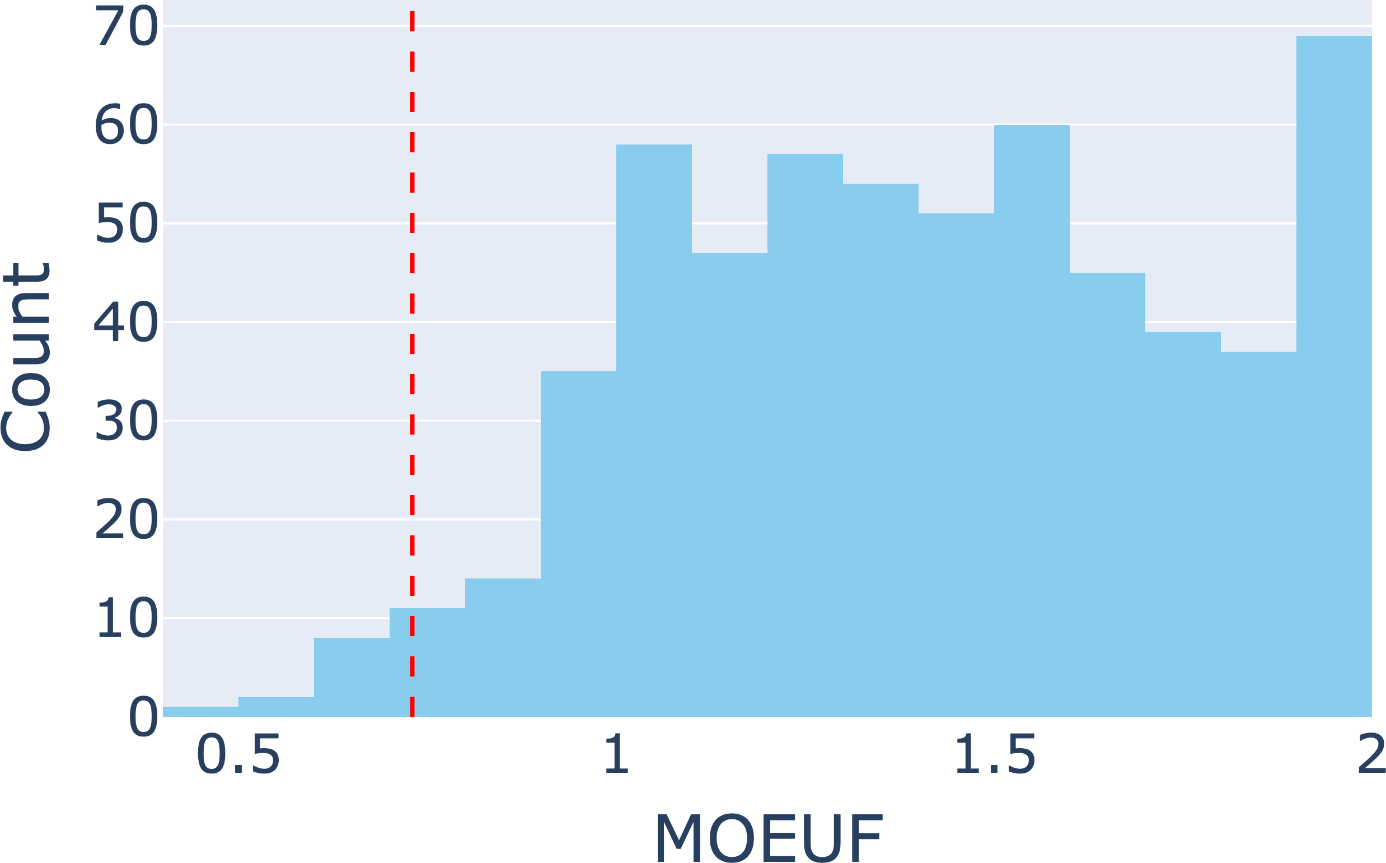


**Supplementary Figure 10:** MOEUF distribution for the uoORF sORF class, calculated using the gnomAD genome data. Decile comparison was performed as introduced previously. The red vertical line (0.73) marks the most constrained decile of the MOEUF value of OEUF scores calculated for the MANE select transcripts of the canonical genes using the gnomAD genome data.


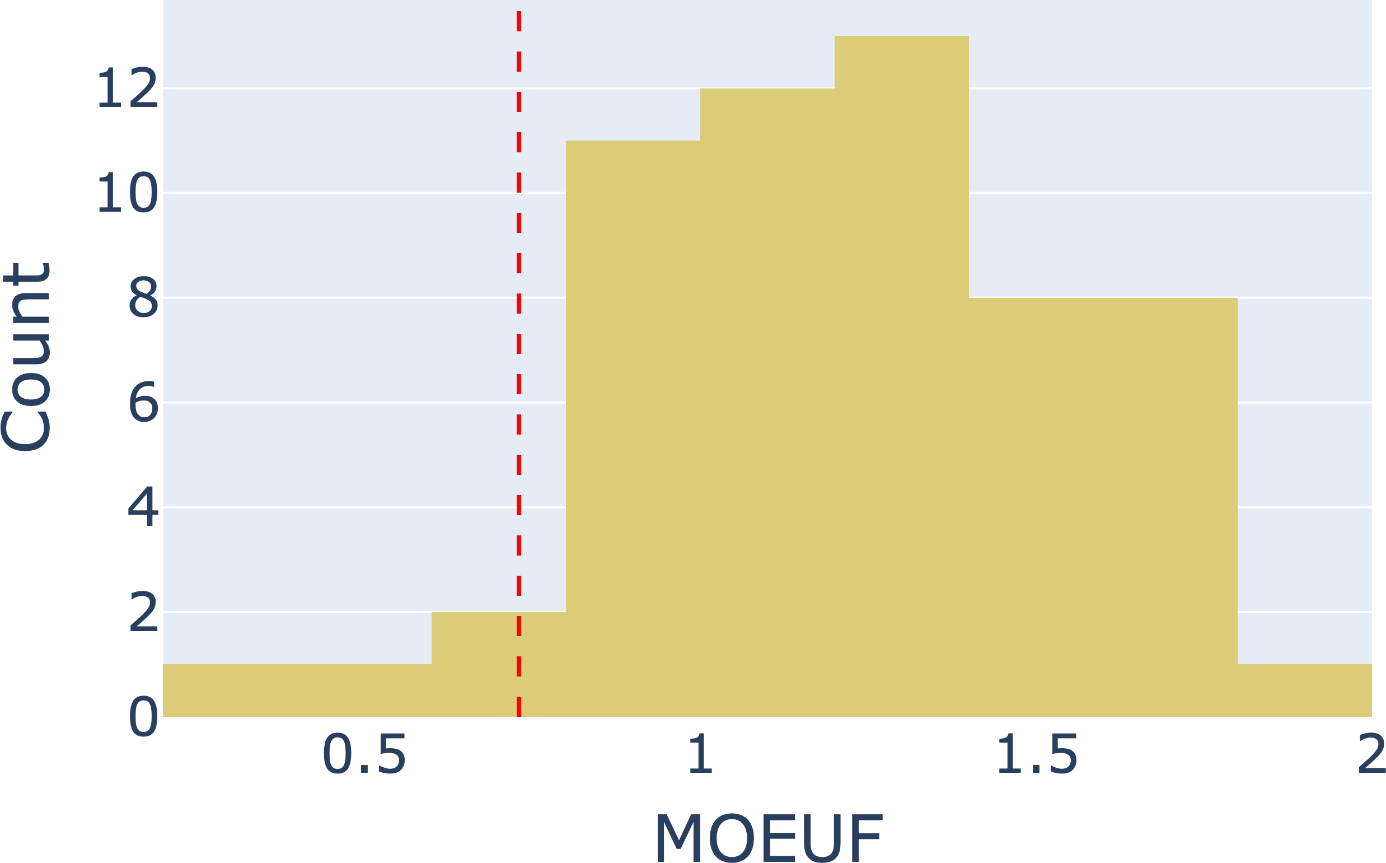


**Supplementary Figure 11:** MOEUF distribution for the doORF sORF class, calculated using the gnomAD genome data. Decile comparison was performed as introduced previously. The red vertical line (0.73) marks the most constrained decile of the MOEUF value of OEUF scores calculated for the MANE select transcripts of the canonical genes using the gnomAD genome data.


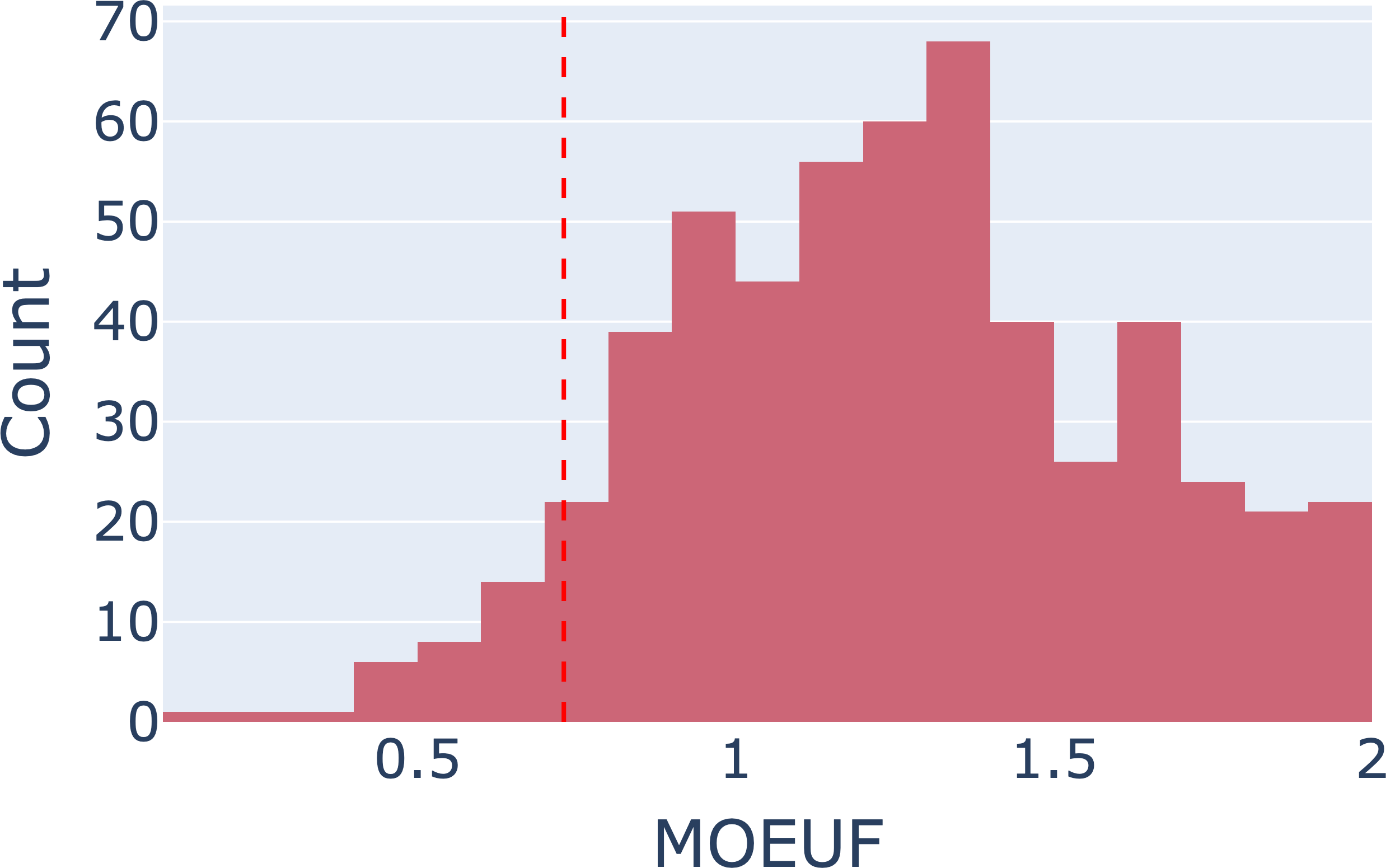


**Supplementary Figure 12:** MOEUF distribution for the intORF sORF class, calculated using the gnomAD genome data. Decile comparison was performed as introduced previously. The red vertical line (0.73) marks the most constrained decile of the MOEUF value of OEUF scores calculated for the MANE select transcripts of the canonical genes using the gnomAD genome data.

**MOEUF distributions for the individual sORF classes (exome data)**

Followingly, we listed the MOEUF distributions for the individual sORF classes using the exome genome.


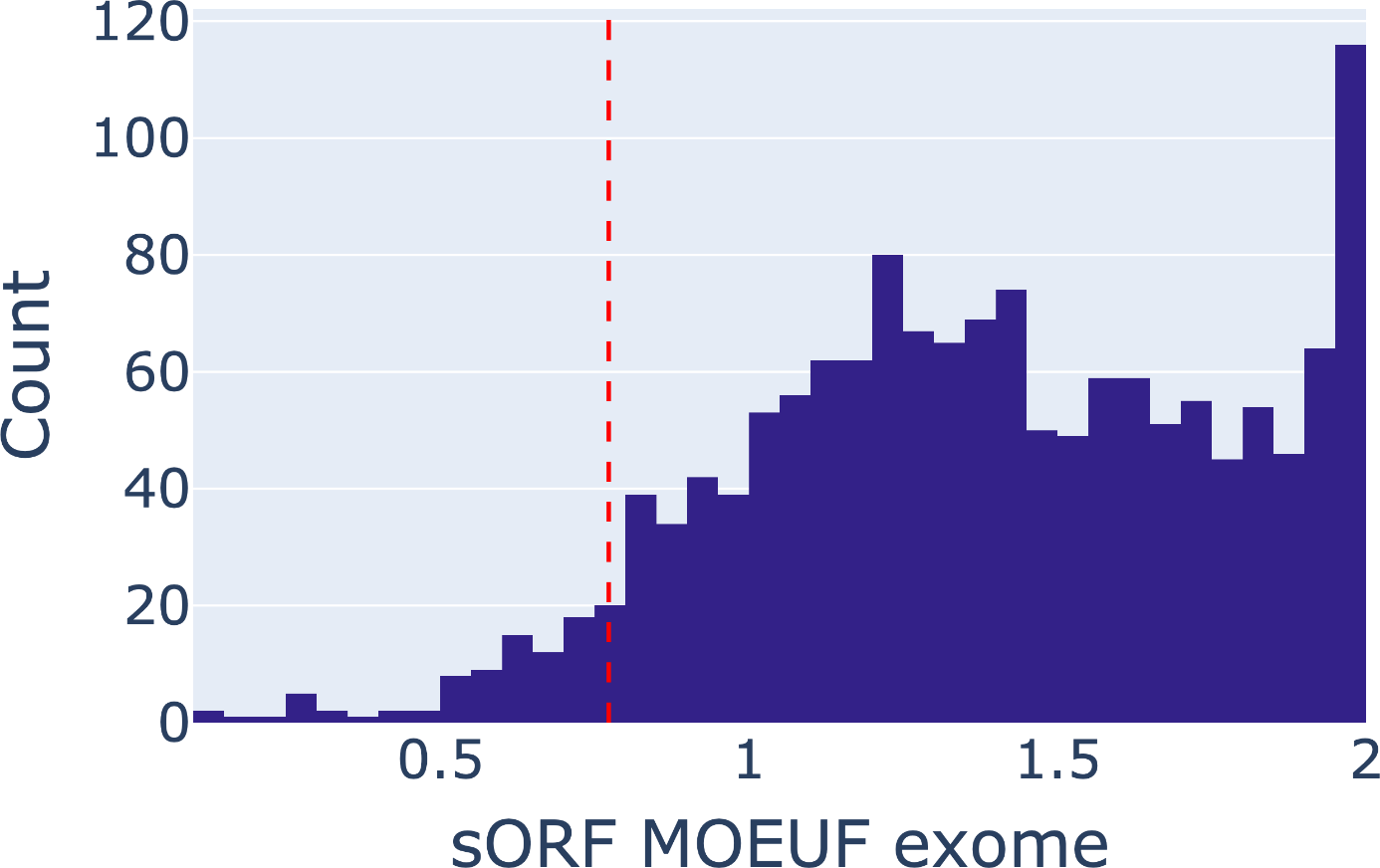


**Supplementary Figure 13:** MOEUF distribution for the uORF sORF class, calculated using the gnomAD exome data. Decile comparison was performed as introduced previously. The red vertical line (0.77) marks the most constrained decile of the MOEUF value of OEUF scores calculated for the MANE select transcripts of the canonical genes using the gnomAD exome data.


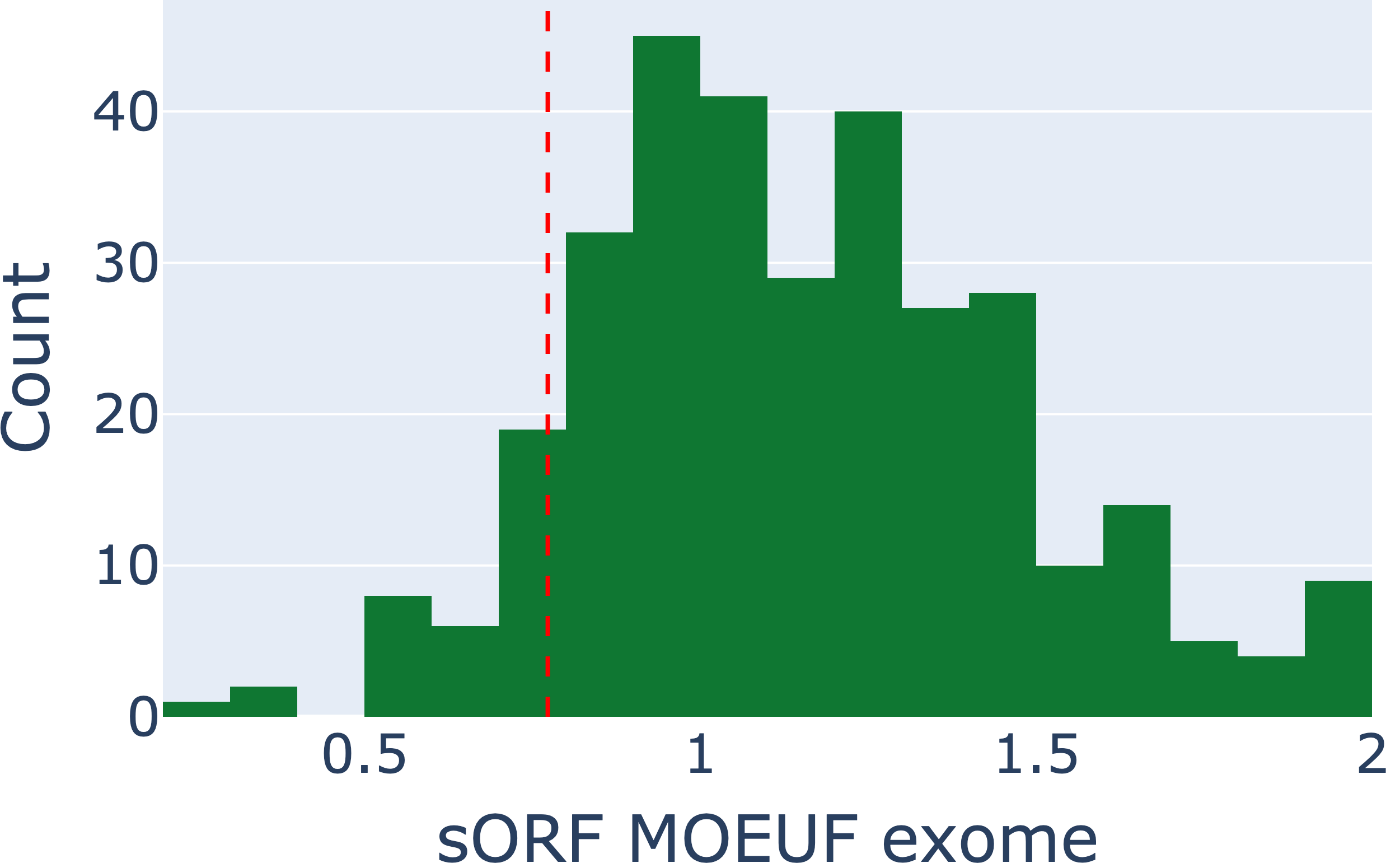


**Supplementary Figure 14:** MOEUF distribution for the dORF sORF class, calculated using the gnomAD exome data. Decile comparison was performed as introduced previously. The red vertical line (0.77) marks the most constrained decile of the MOEUF value of OEUF scores calculated for the MANE select transcripts of the canonical genes using the gnomAD exome data.


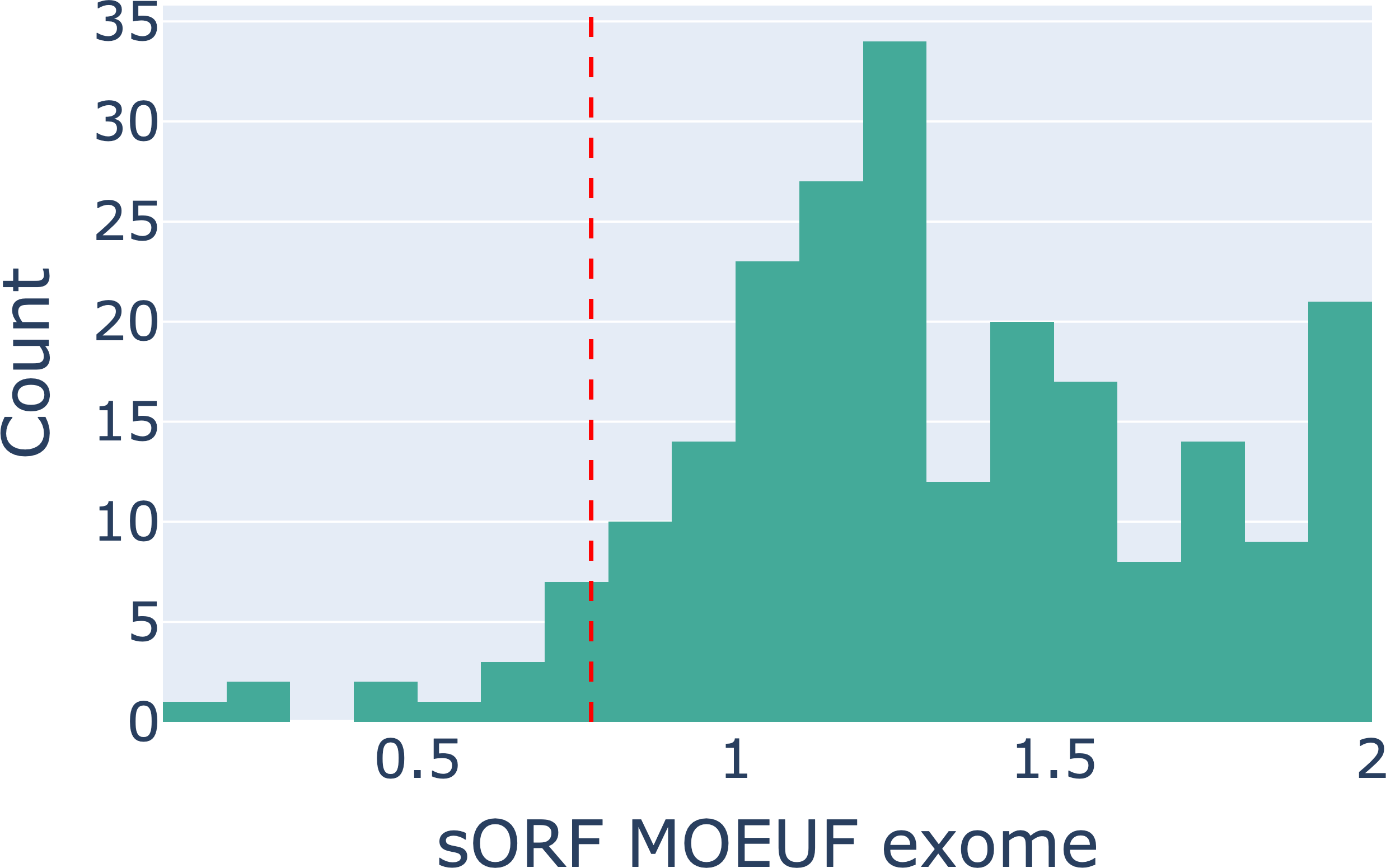


**Supplementary Figure 15:** MOEUF distribution for the dORF sORF class, calculated using the gnomAD exome data. Decile comparison was performed as introduced previously. The red vertical line (0.77) marks the most constrained decile of the MOEUF value of OEUF scores calculated for the MANE select transcripts of the canonical genes using the gnomAD exome data.


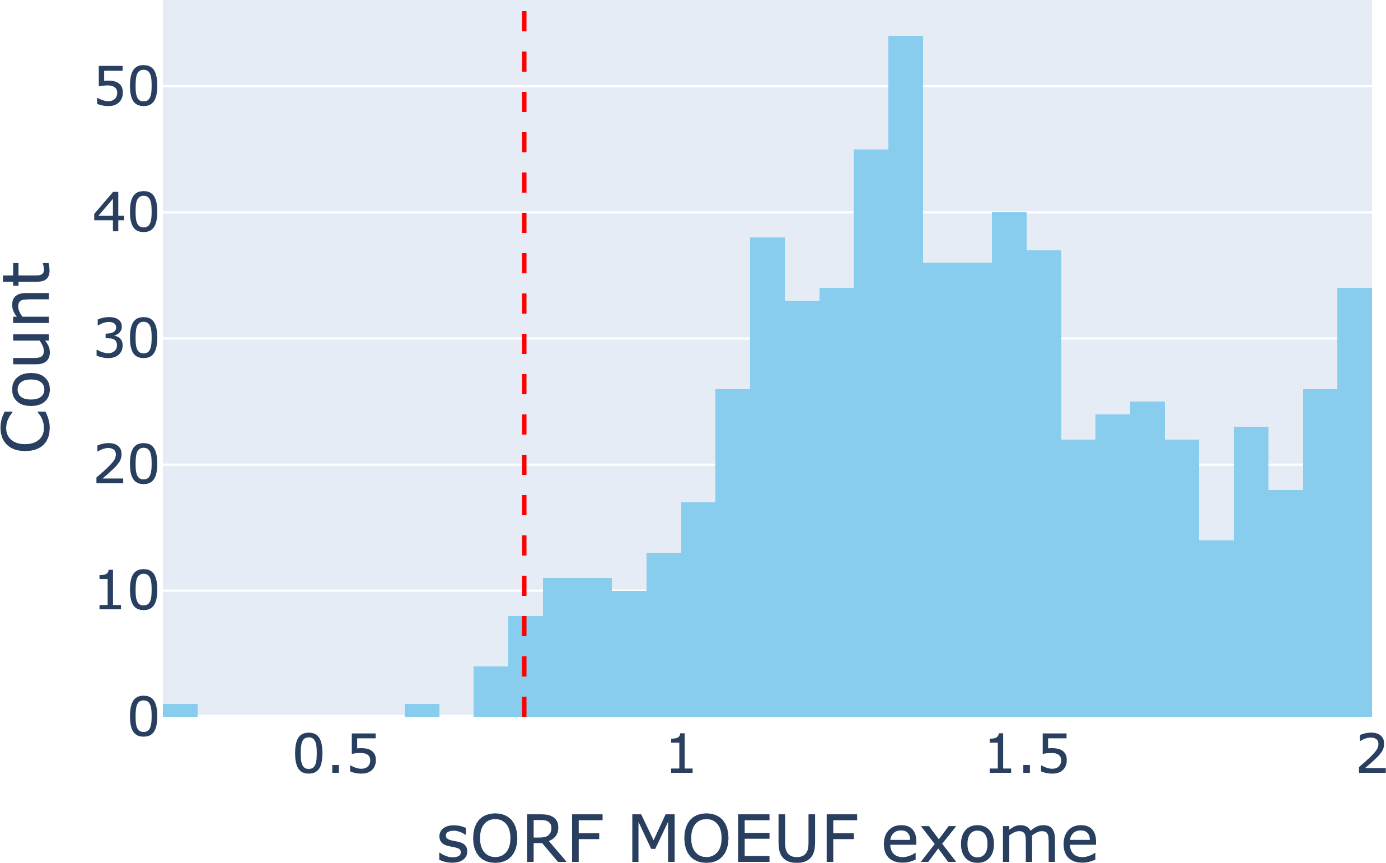


**Supplementary Figure 16:** MOEUF distribution for the dORF sORF class, calculated using the gnomAD exome data. Decile comparison was performed as introduced previously. The red vertical line (0.77) marks the most constrained decile of the MOEUF value of OEUF scores calculated for the MANE select transcripts of the canonical genes using the gnomAD exome data.


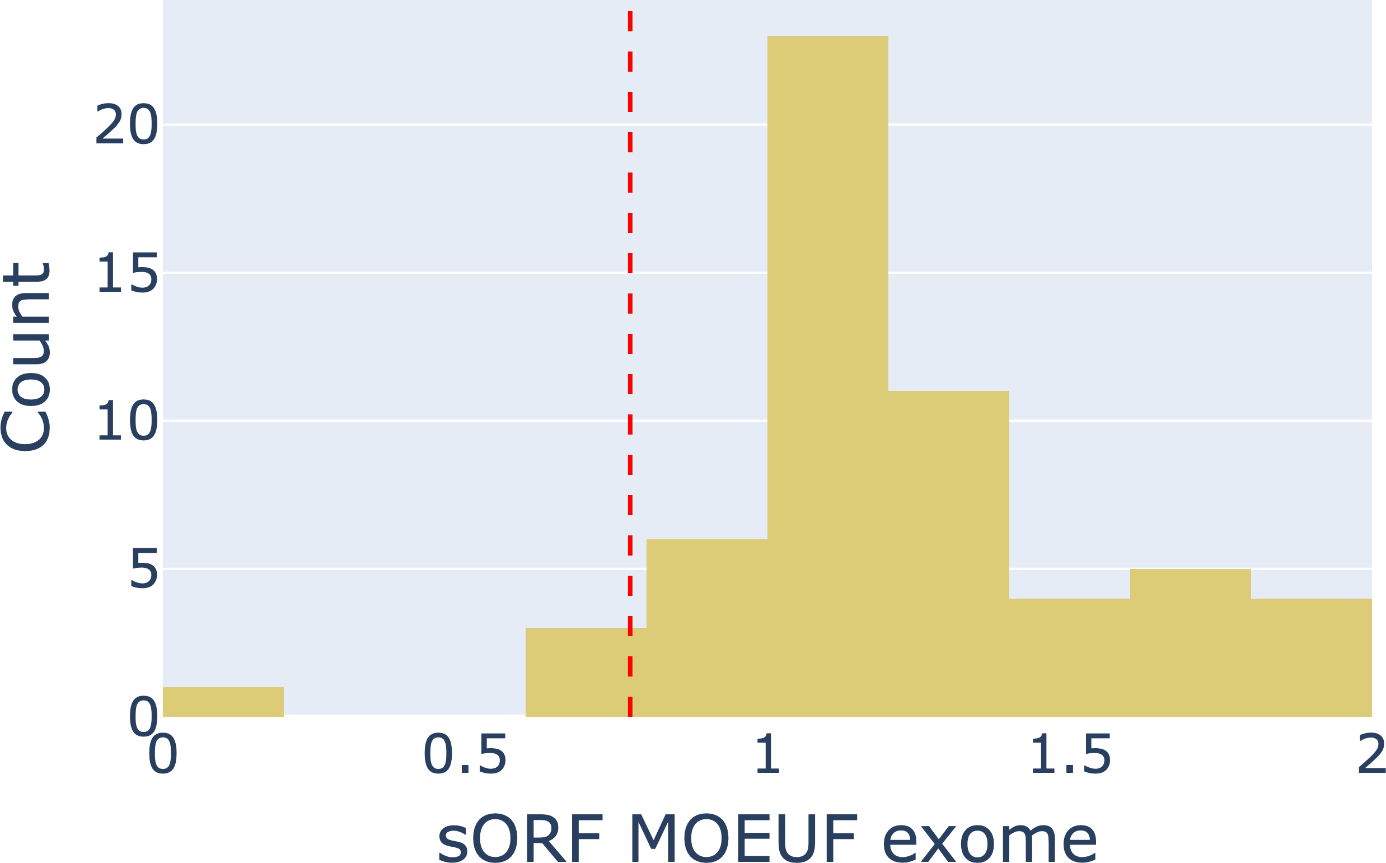


**Supplementary Figure 17:** MOEUF distribution for the dORF sORF class, calculated using the gnomAD exome data. Decile comparison was performed as introduced previously. The red vertical line (0.77) marks the most constrained decile of the MOEUF value of OEUF scores calculated for the MANE select transcripts of the canonical genes using the gnomAD exome data.


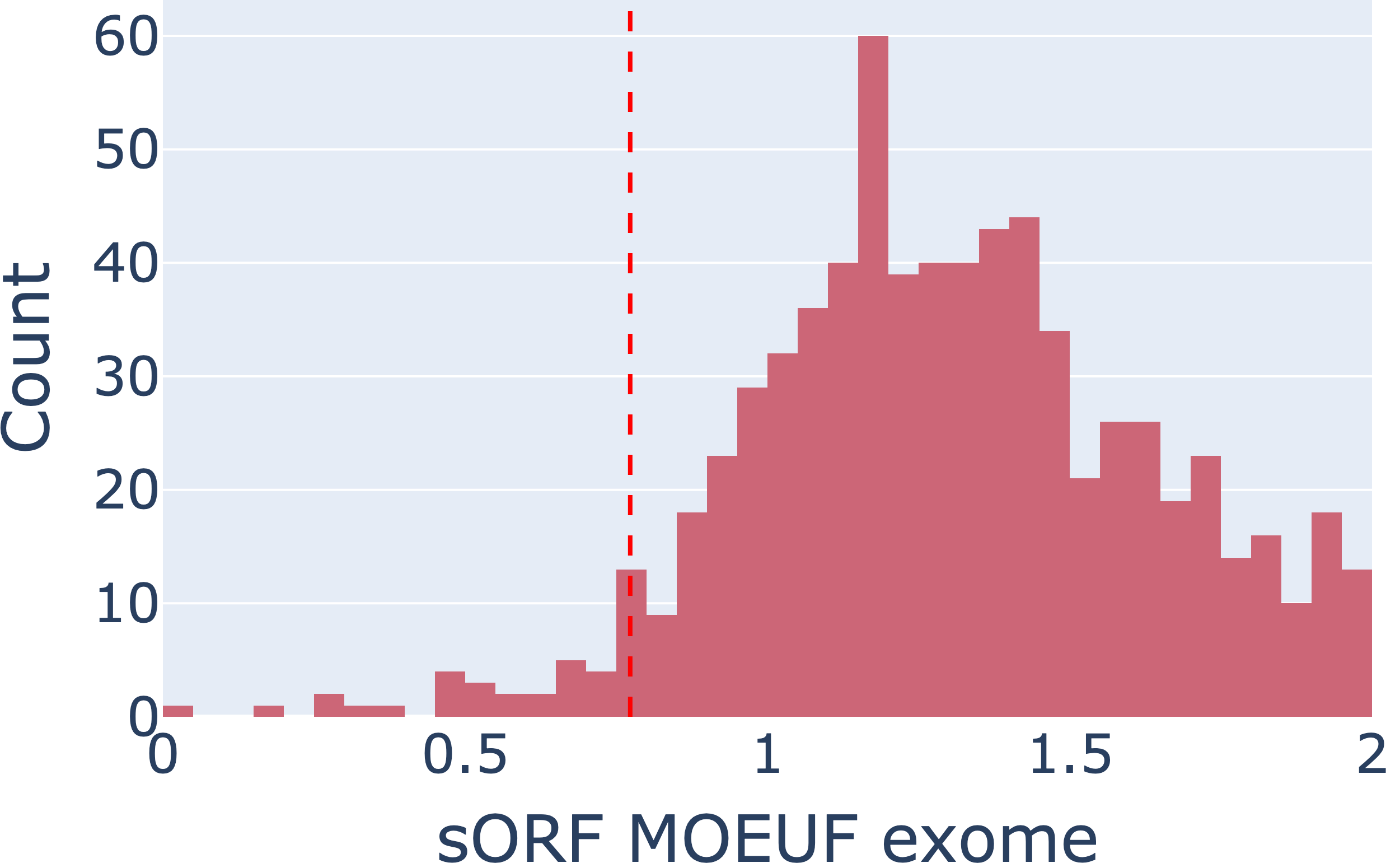


**Supplementary Figure 18:** MOEUF distribution for the dORF sORF class, calculated using the gnomAD exome data. Decile comparison was performed as introduced previously. The red vertical line (0.77) marks the most constrained decile of the MOEUF value of OEUF scores calculated for the MANE select transcripts of the canonical genes using the gnomAD exome data.

**Individual sORF class comparison with the Gnocchi score**

Additionally, to the summary plot found in the article, we provide individual plots for comparison, in which we compare the SNVOEUF score of the induvial sORF classes with the Gnocchi Score.


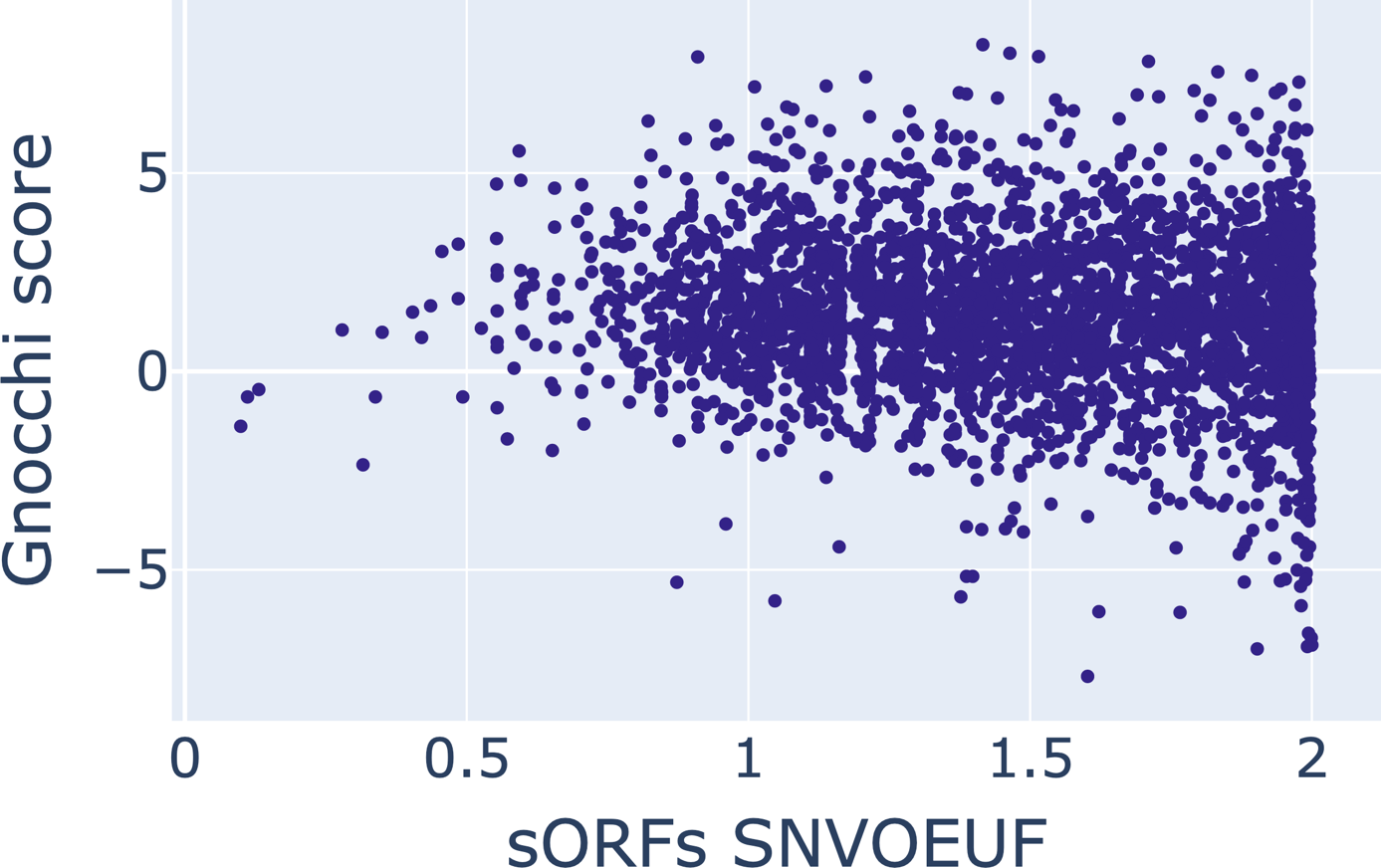


**Supplementary Figure 19:** Comparison between SNVOEUF of uORFs calculated using gnomAD genomes and the Gnocchi Score (Kendall Rank Correlation Coefficient =-0.06, p < 0.001).


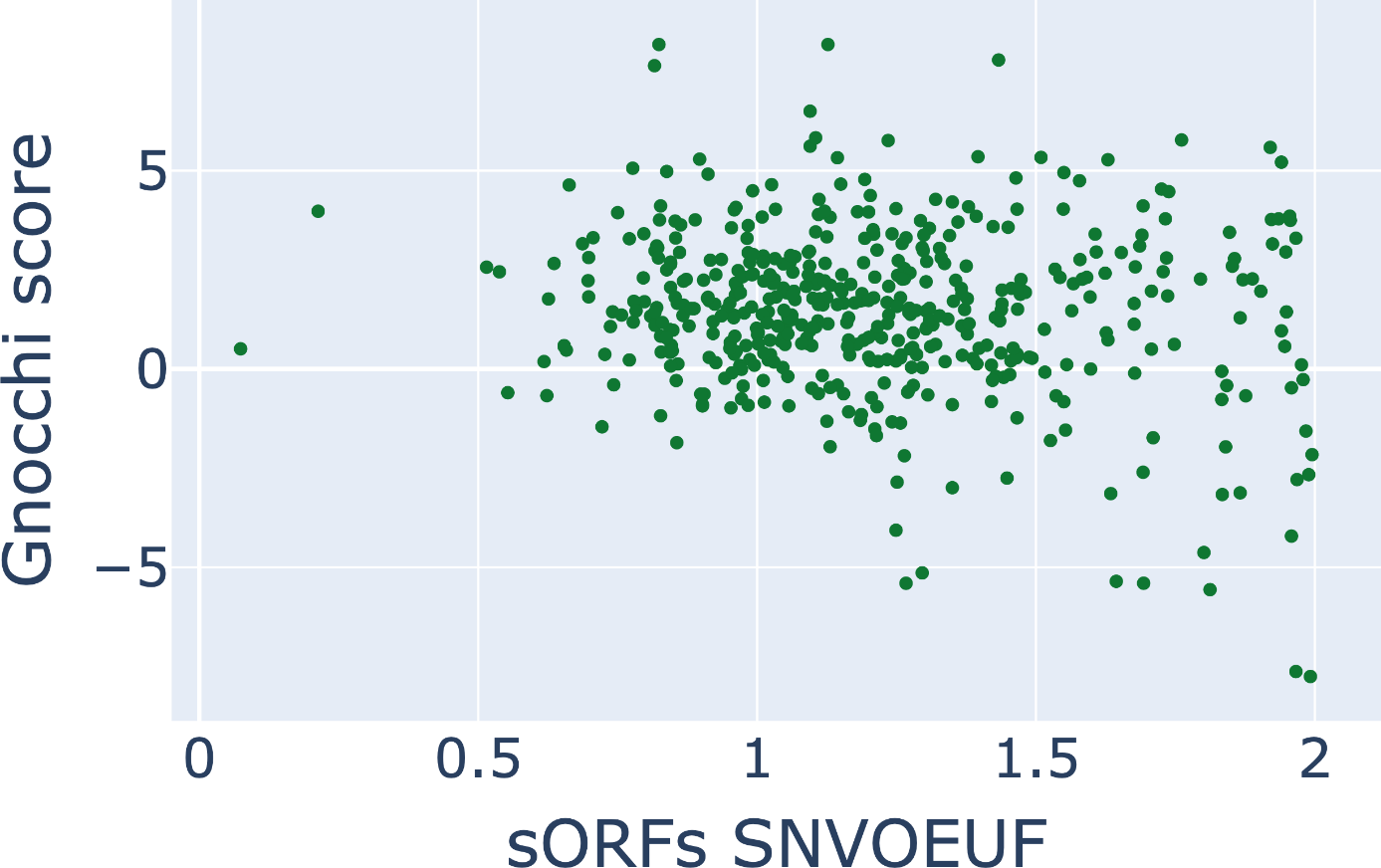


**Supplementary Figure 20:** Comparison between SNVOEUF of dORFs calculated using gnomAD genomes and the Gnocchi Score (Kendall Rank Correlation Coefficient = -0.051, p = 0.09).


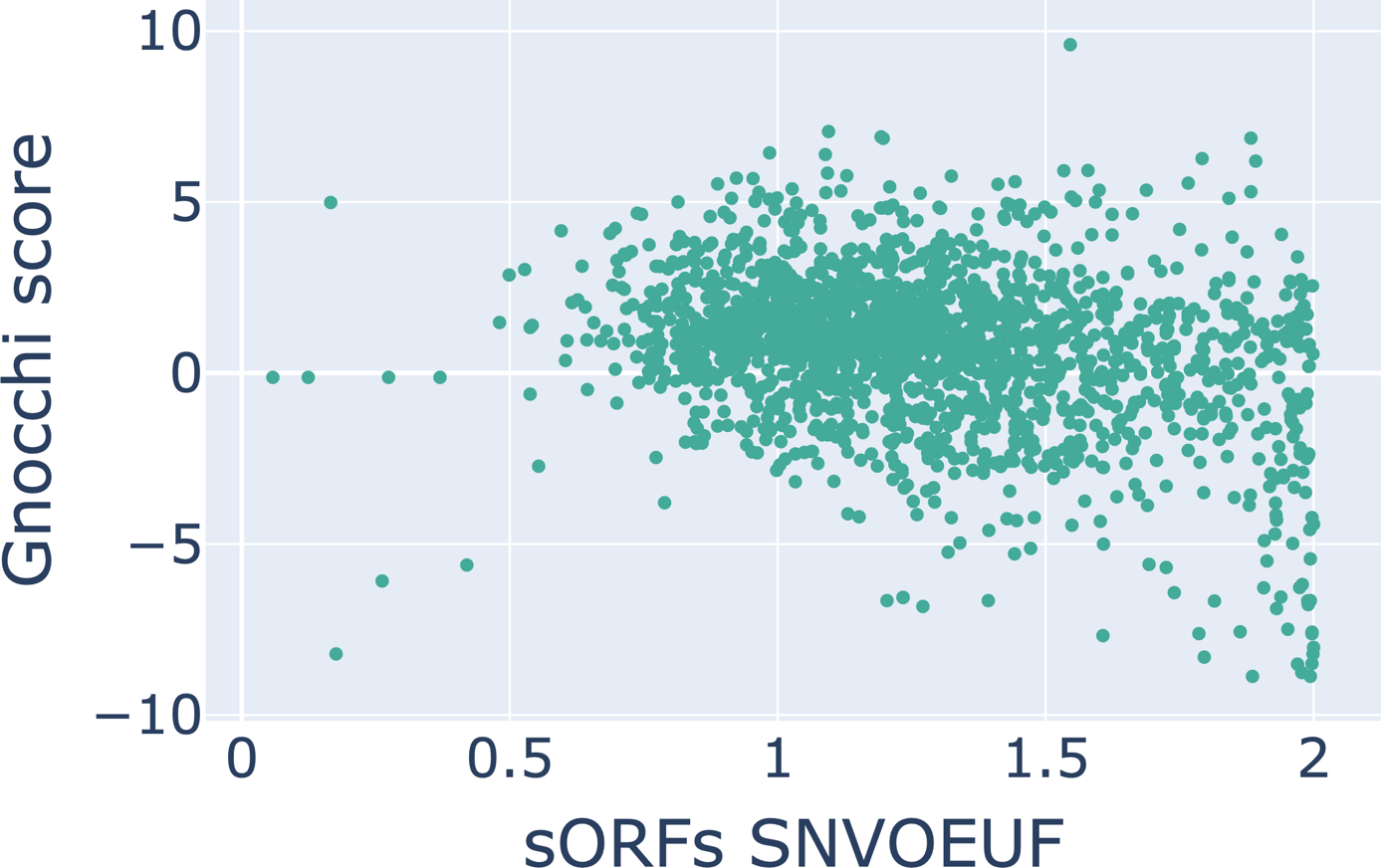


**Supplementary Figure 21:** Comparison between SNVOEUF of sORFs located on lncRNAs calculated using gnomAD genomes and the Gnocchi Score (Kendall Rank Correlation Coefficient = -0.16, p < 0.001).


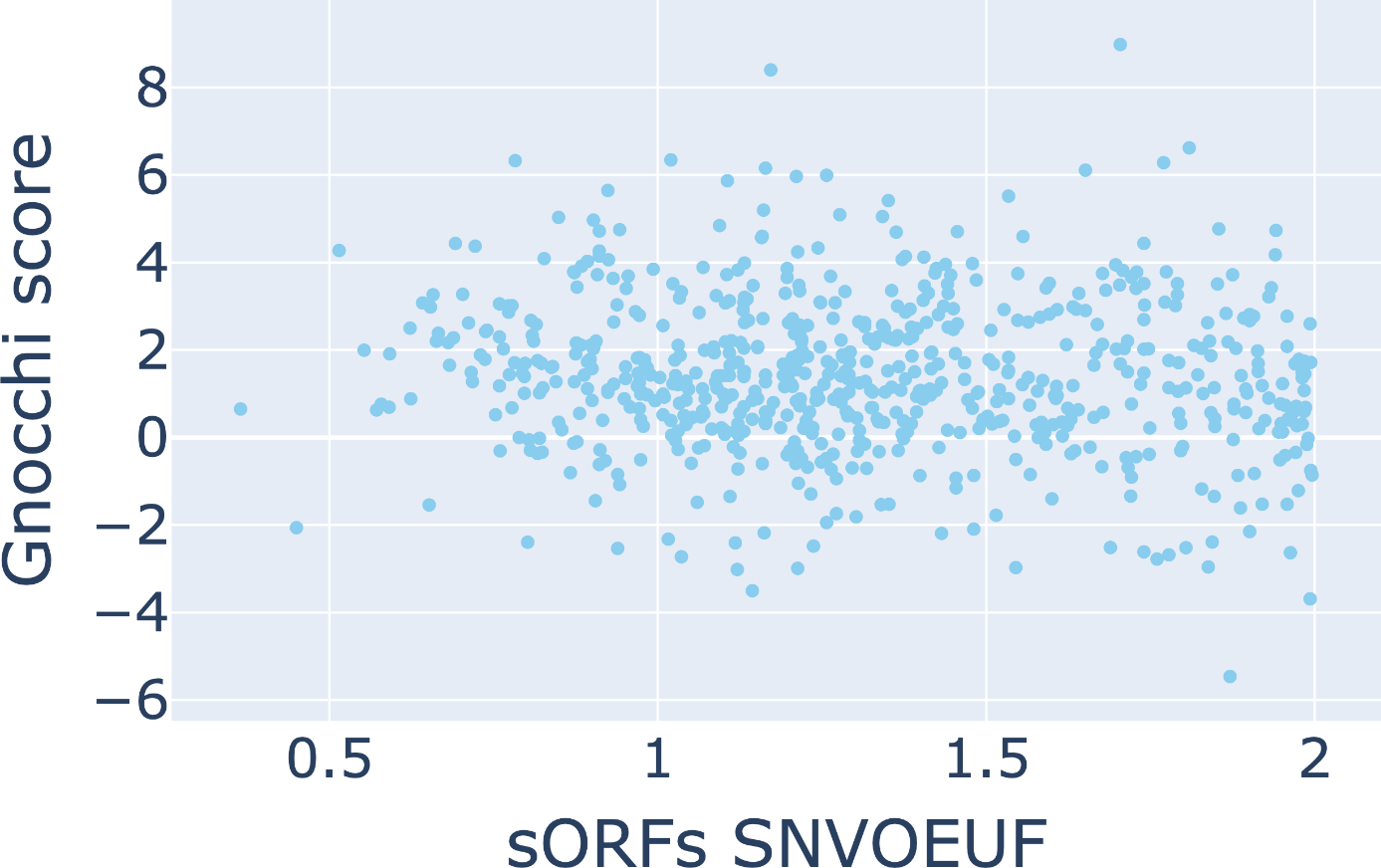


**Supplementary Figure 22:** Comparison between SNVOEUF of uoORFs calculated using gnomAD genomes and the Gnocchi Score (Kendall Rank Correlation Coefficient = -0.06, p = 0.02).


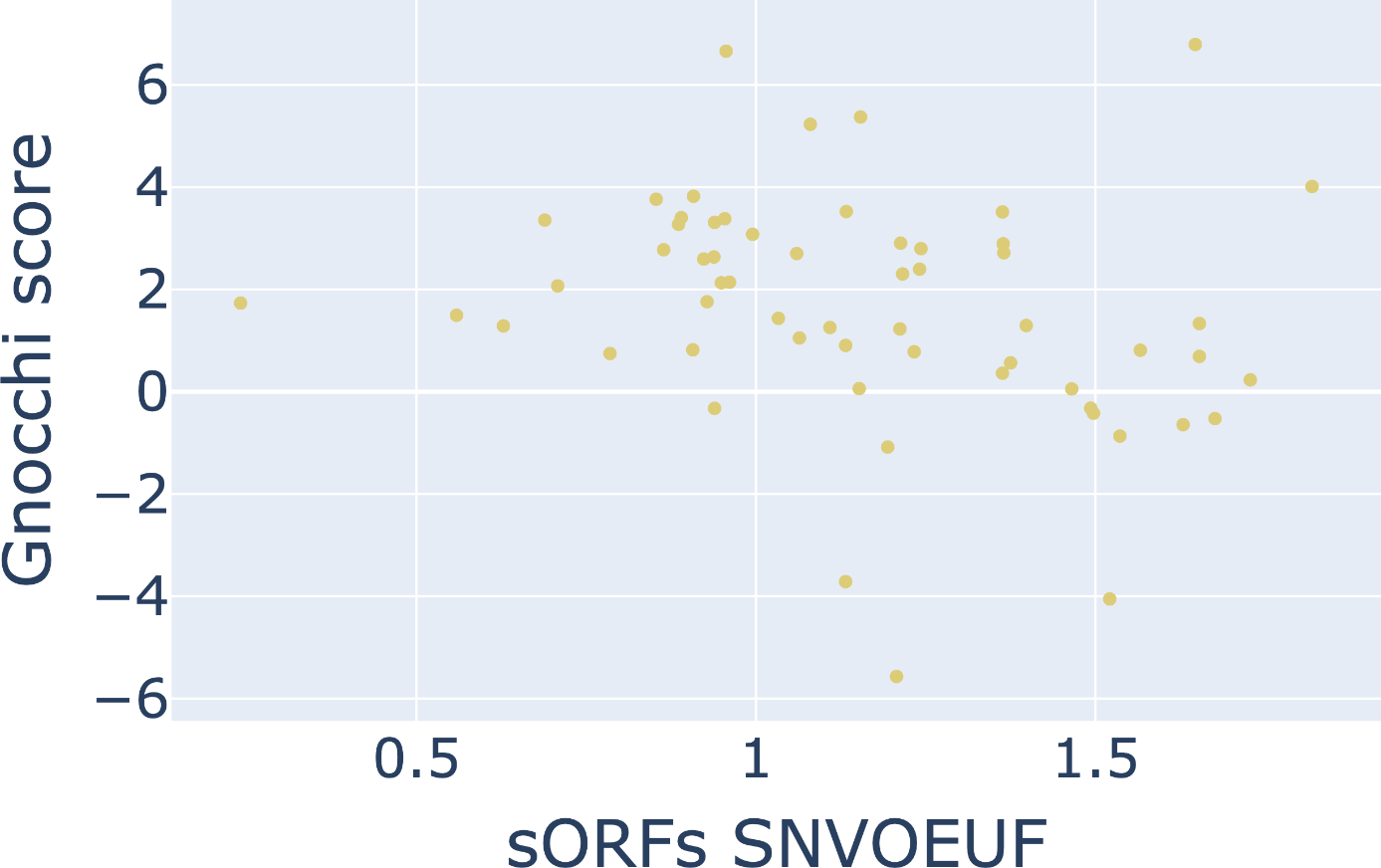


**Supplementary Figure 23:** Comparison between SNVOEUF of doORFs calculated using gnomAD genomes and the Gnocchi Score (Kendall Correlation Coefficient = -0.23, p = 0.12).


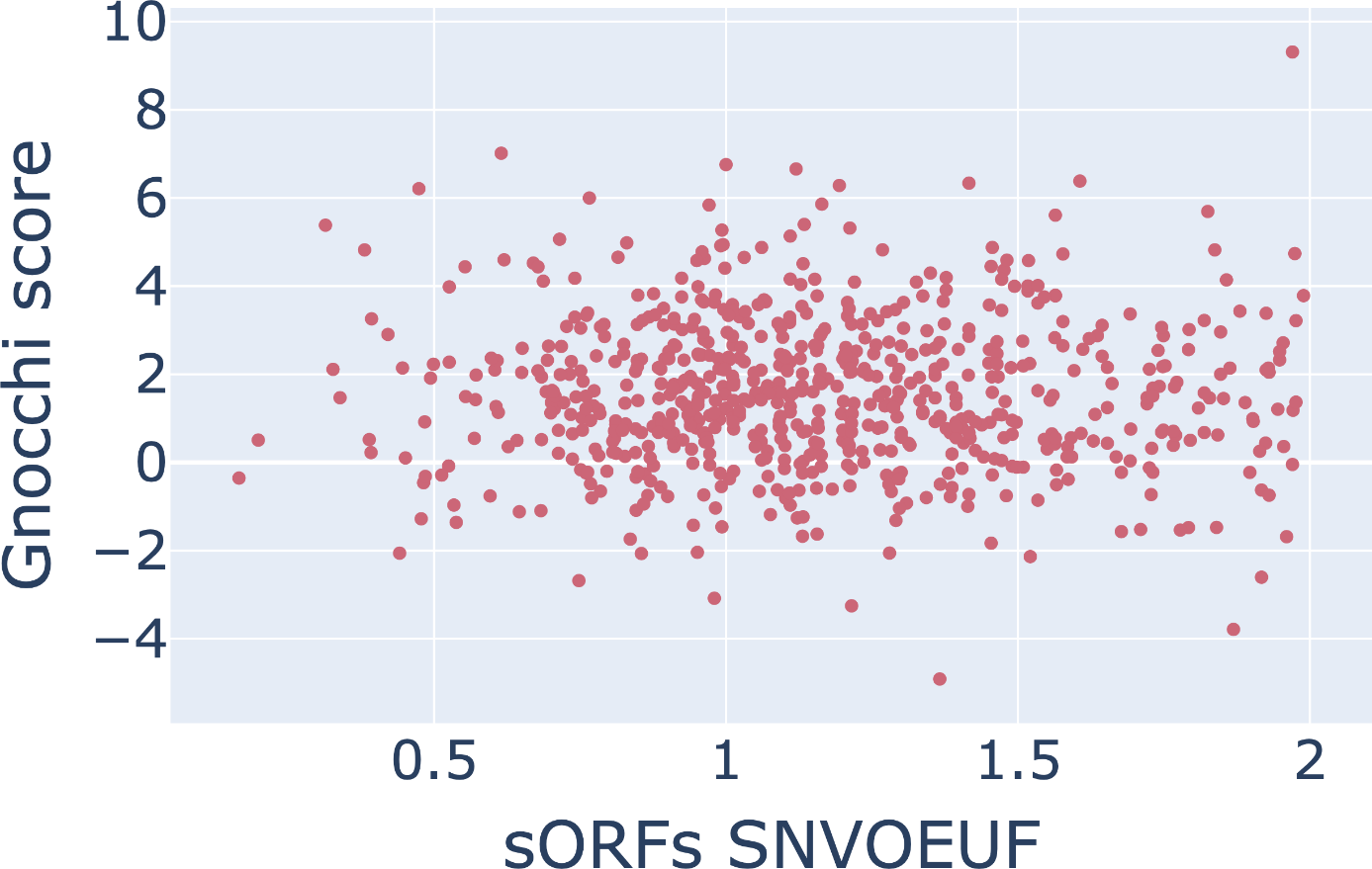


**Supplementary Figure 24:** Comparison between SNVOEUF of intORFs calculated using gnomAD genomes and the Gnocchi Score (Kendall Correlation Coefficient = -0.006, p=0.82).

**Individual sORF class comparison with the MOEUF of neighboured genes**

Additionally, to the summary plot found in the article, we provide individual plots for comparison, in which we compare the MOEUF score of the induvial sORF classes with the MOEUF scores of neighboured genes.


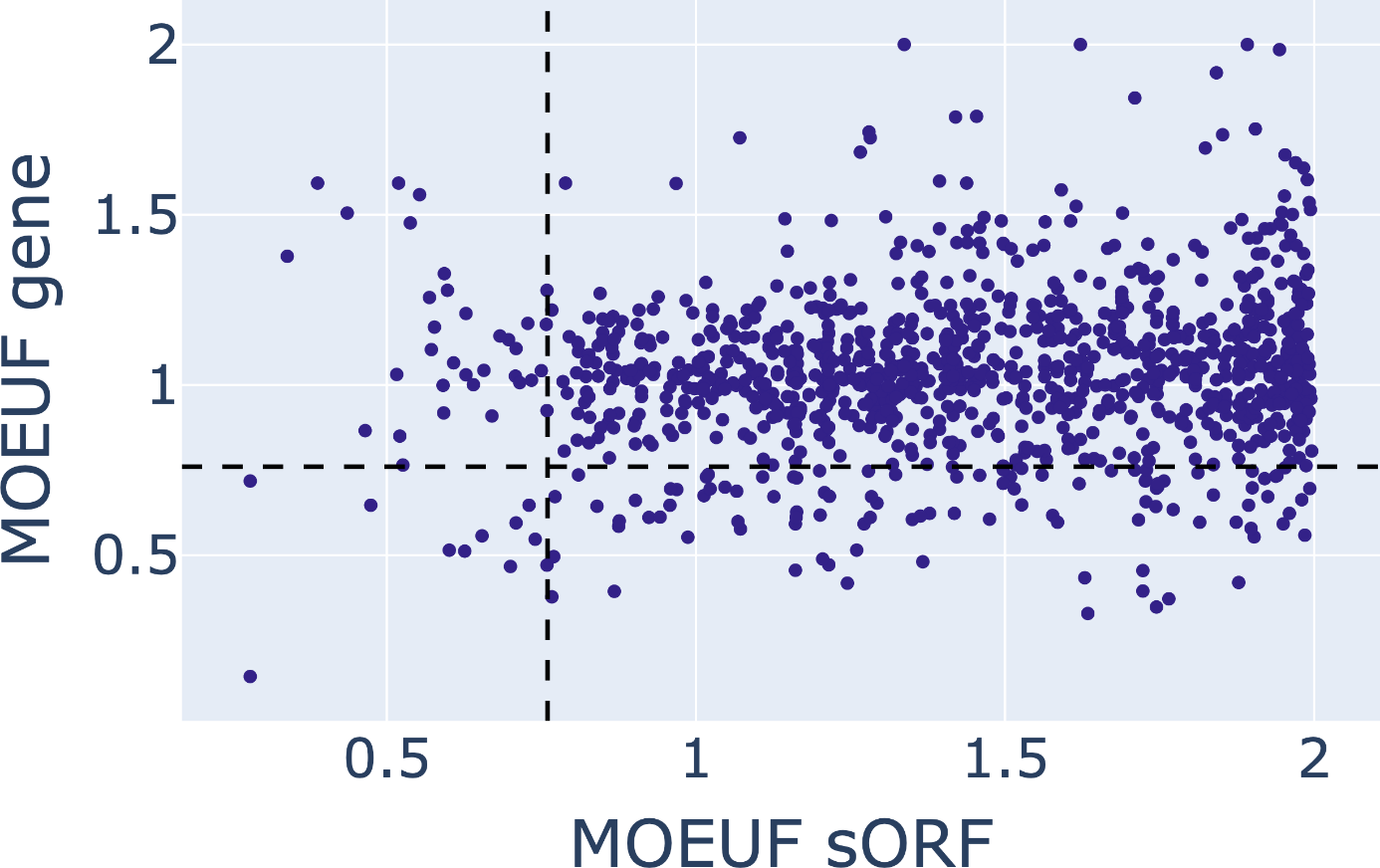


**Supplementary Figure 25:** Comparison between MOEUF of uORFs and neighboured genes calculated using gnomAD genomes (Kendall Rank Correlation Coefficient = 0.066, p < 0.001).


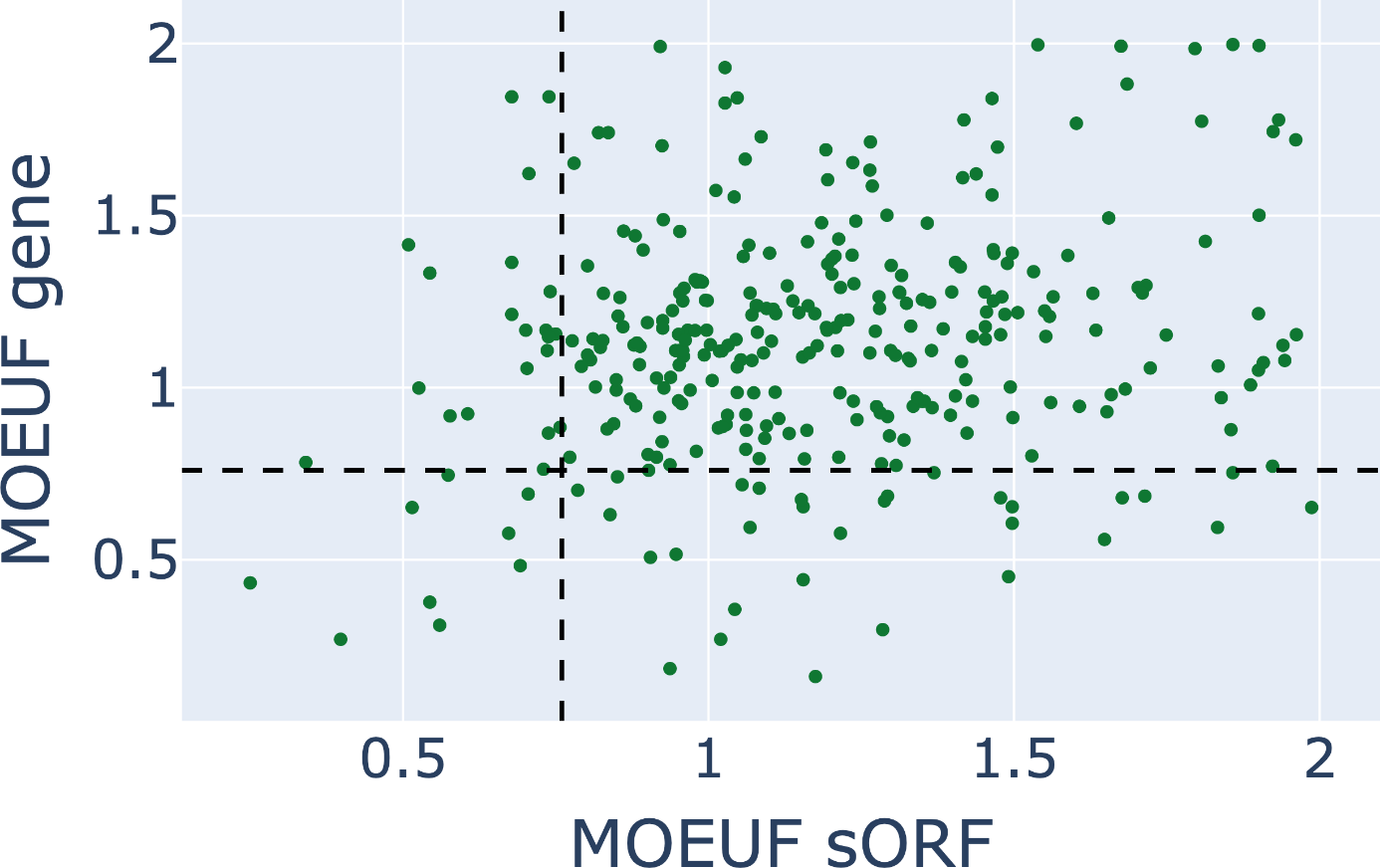


**Supplementary Figure 26:** Comparison between MOEUF of dORFs and neighboured genes calculated using gnomAD genomes (Kendall Rank Correlation Coefficient = 0.105. p = 0.005).


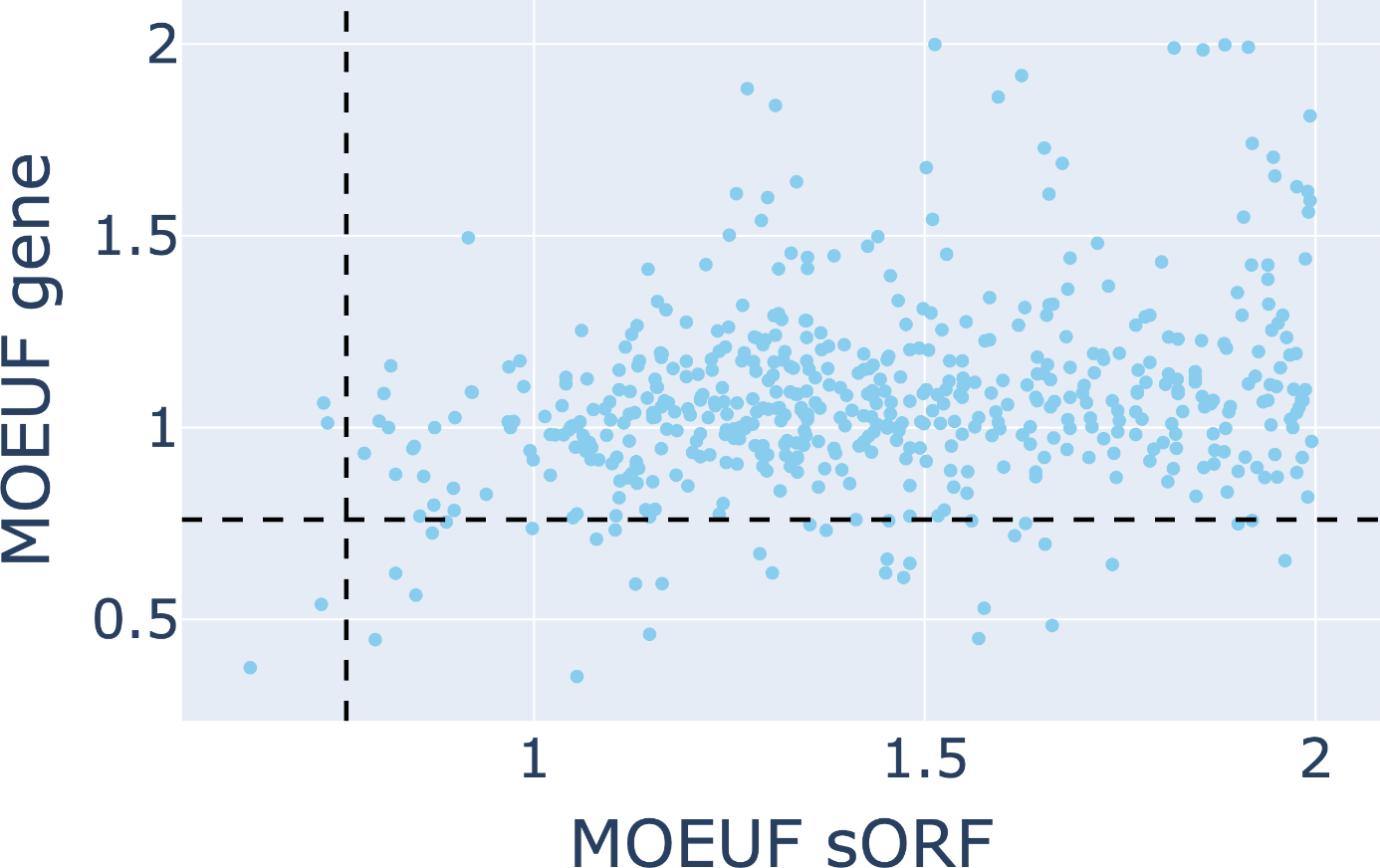


**Supplementary Figure 27:** Comparison between MOEUF of uoORFs and neighboured genes calculated using gnomAD genomes (Kendall Rank Correlation Coefficient = 0.158, p < 0.001).


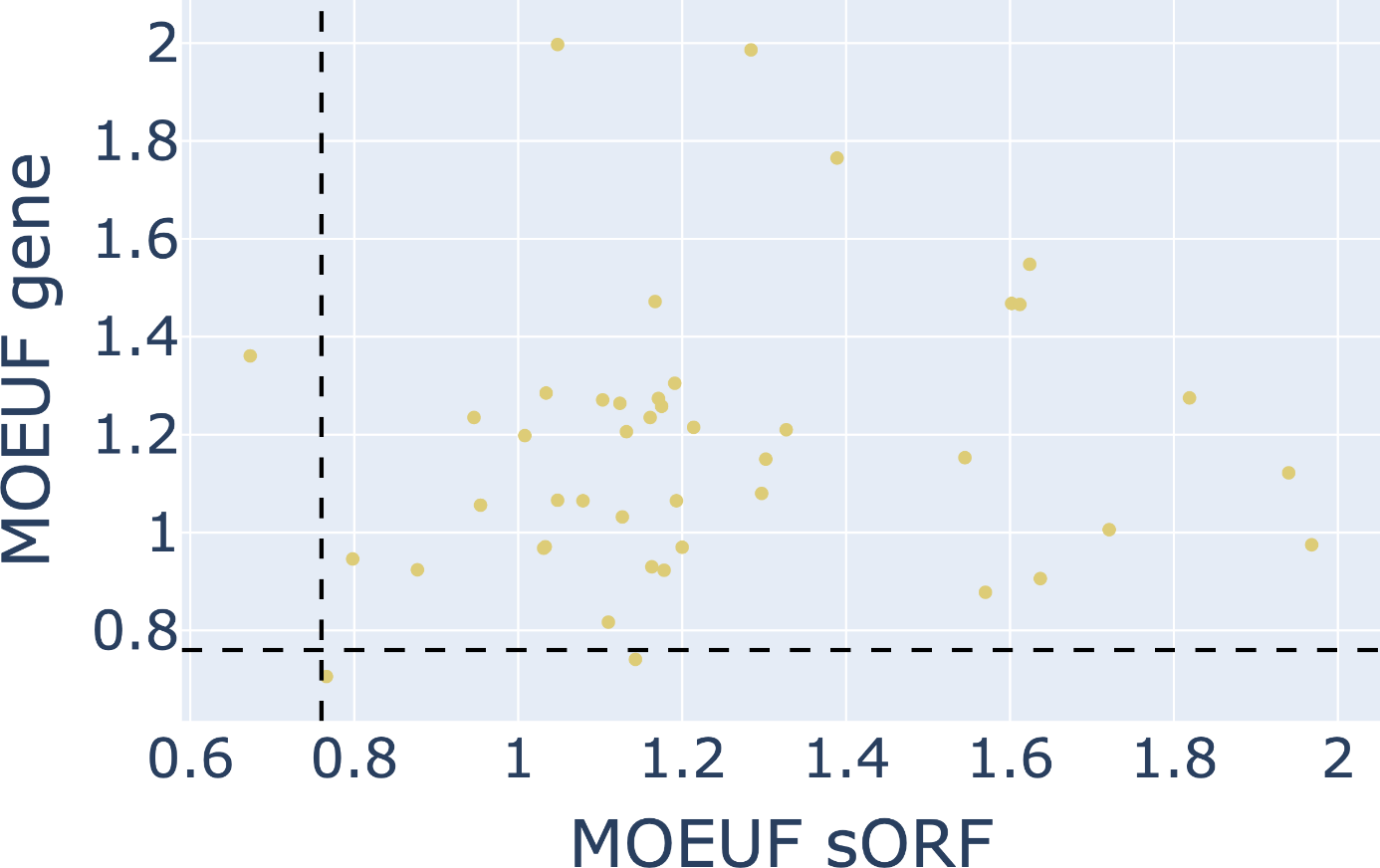


**Supplementary Figure 28:** Comparison between MOEUF of doORFs and neighboured genes calculated using gnomAD genomes (Kendall Rank Correlation Coefficient = 0.118, p = 0.26).


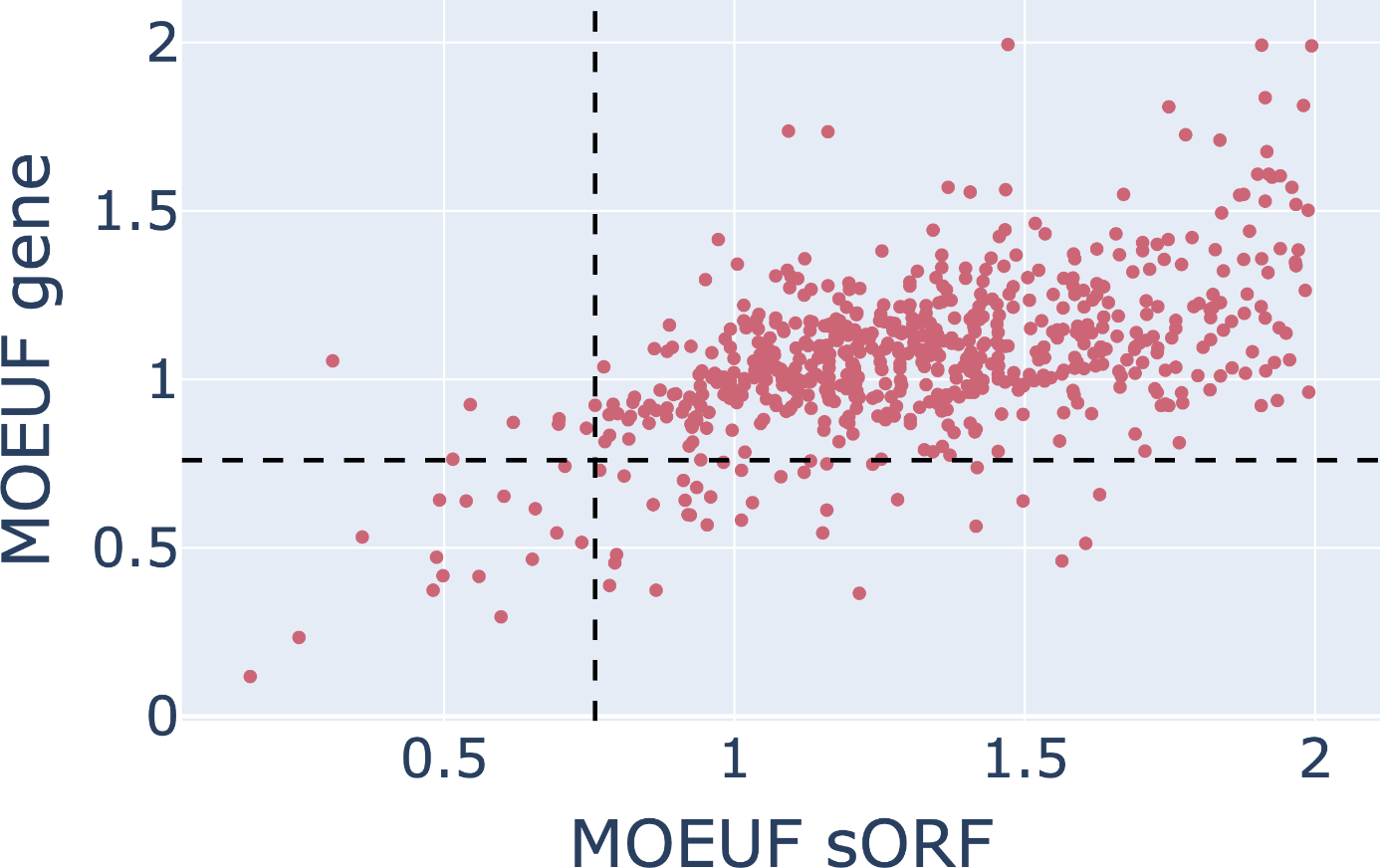


**Supplementary Figure 29:** Comparison between MOEUF of intORFs and neighboured genes calculated using gnomAD genomes (Kendall Rank correlation = 0.35, p < 0.001).

**
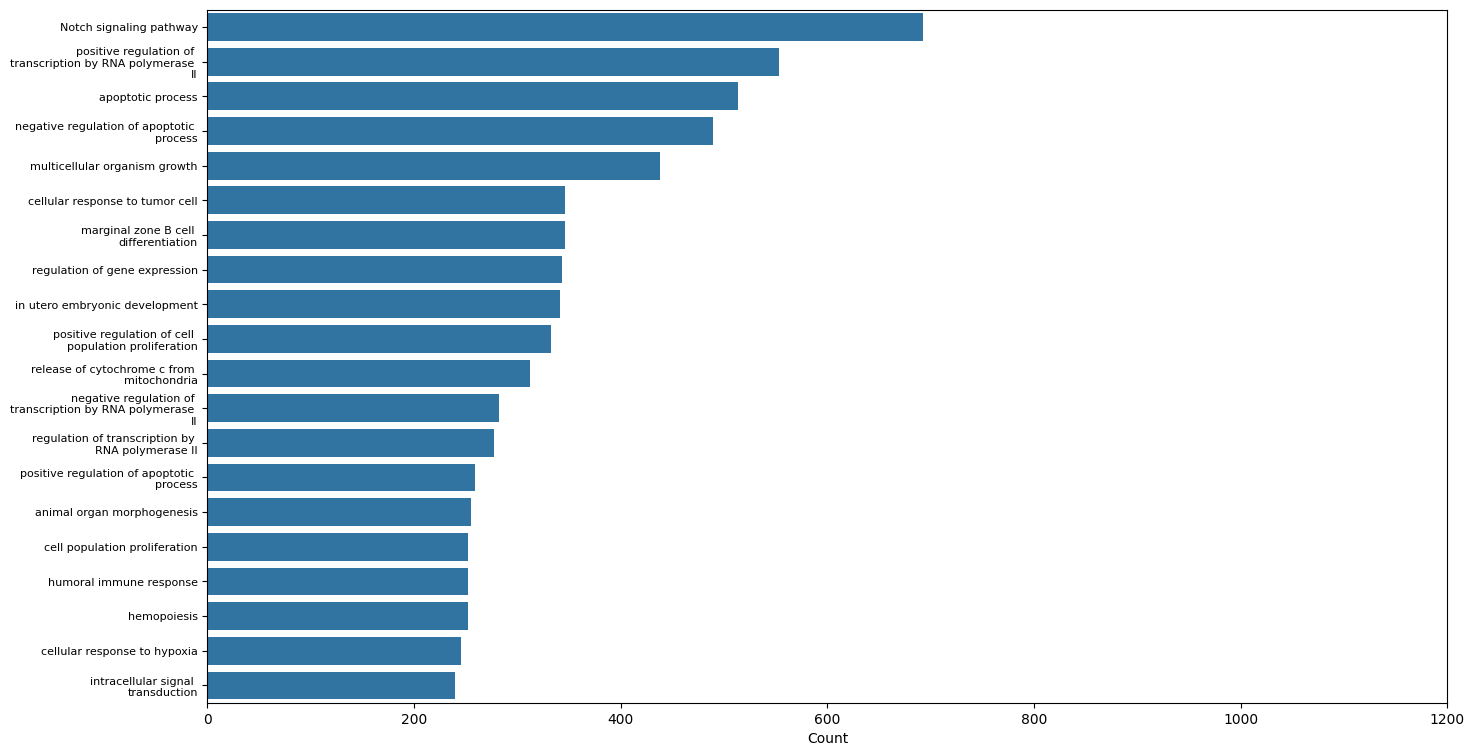
 Supplementary Figure 30:** Top 20 GO terms by count of genes neighboring the highly constrained sORFs for the GO domain – Biological Process.


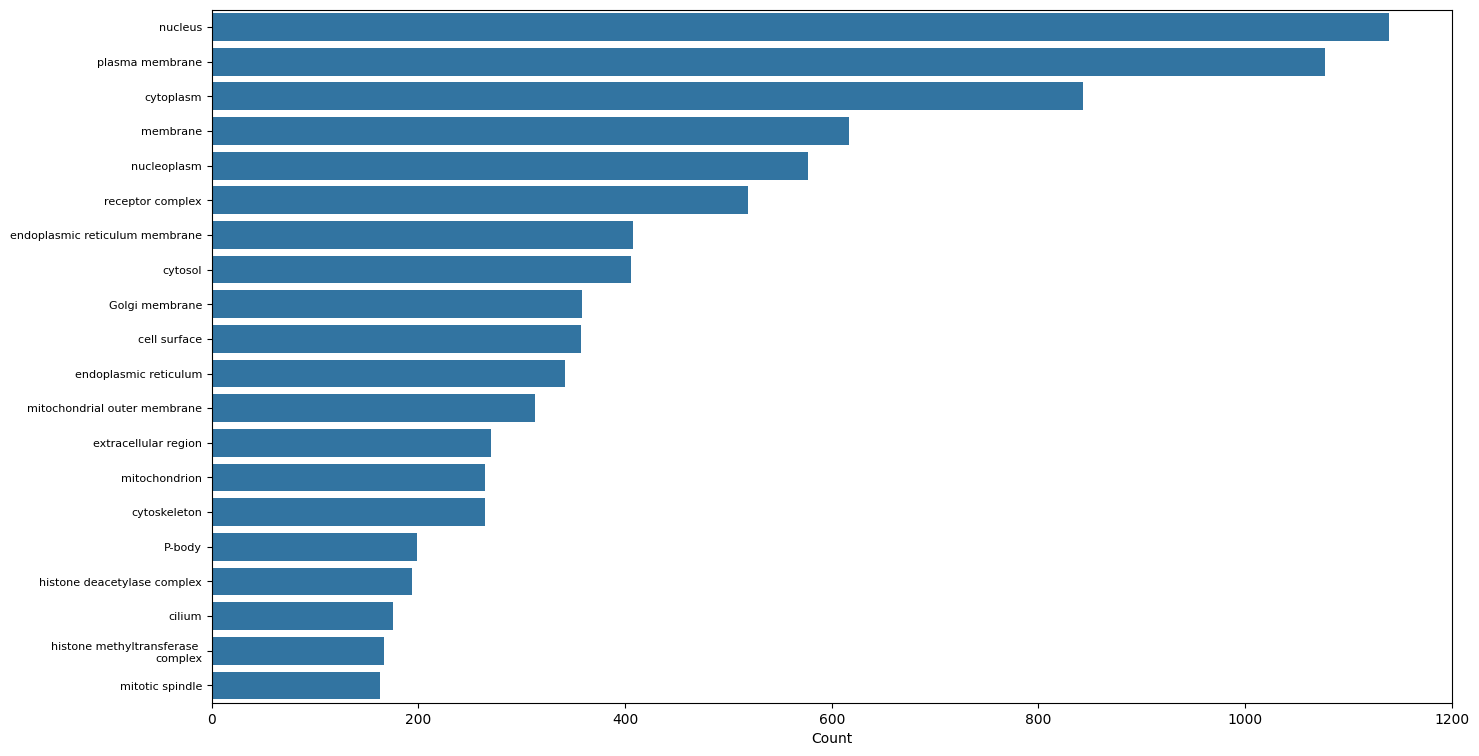


**Supplementary Figure 31:** Top 20 GO terms by count of genes neighboring the highly constrained sORFs for the GO domain – Cellular Component.

**
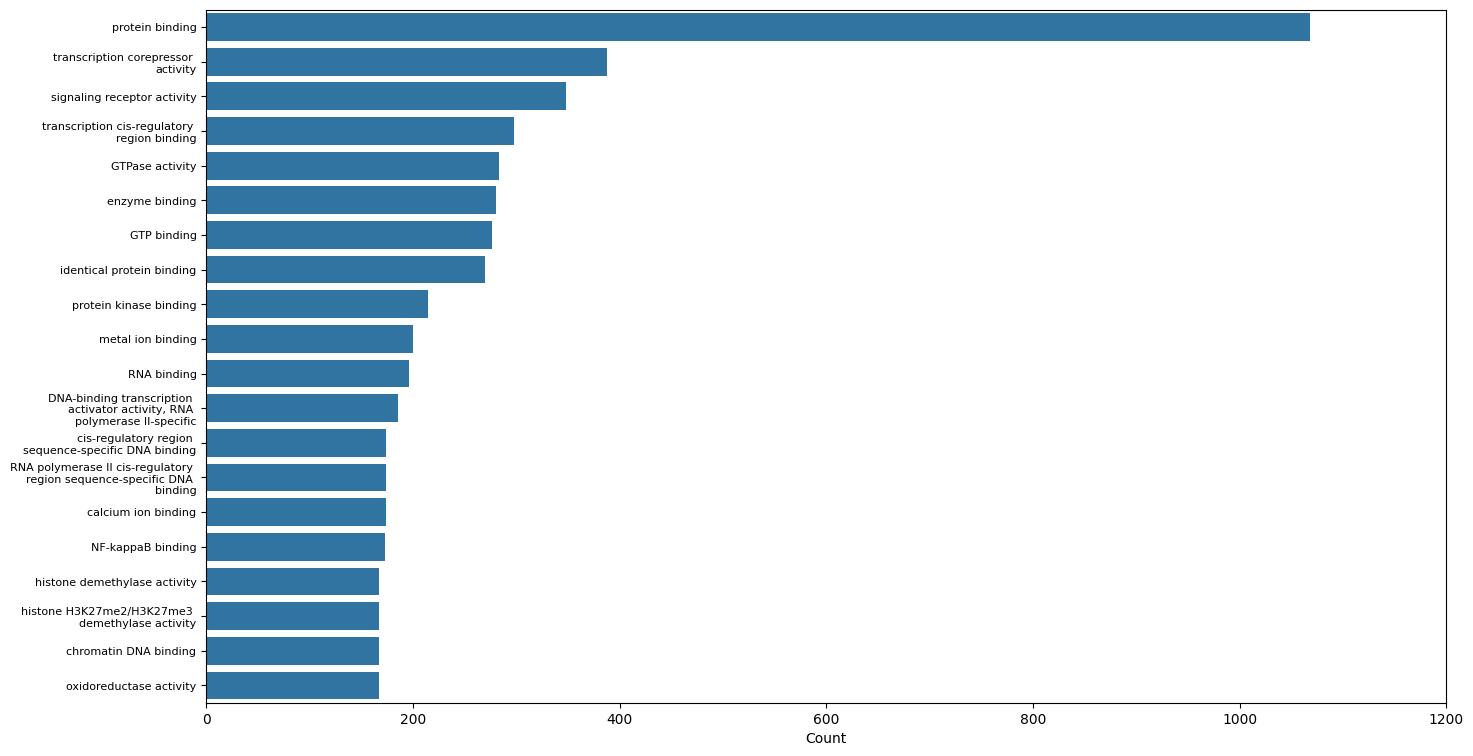
 Supplementary Figure 32:** Top 20 GO terms by count of genes neighboring the highly constrained sORFs for the GO domain – Cellular Component.
